# Repeating patterns: Predictive processing suggests an aesthetic learning role of the basal ganglia in repetitive stereotyped behaviors

**DOI:** 10.3389/fpsyg.2022.930293

**Published:** 2022-09-08

**Authors:** Blanca T. M. Spee, Ronald Sladky, Joerg Fingerhut, Alice Laciny, Christoph Kraus, Sidney Carls-Diamante, Christof Brücke, Matthew Pelowski, Marco Treven

**Affiliations:** ^1^Vienna Cognitive Science Hub, University of Vienna, Vienna, Austria; ^2^Department of Neurology, Center of Expertise for Parkinson and Movement Disorders, Radboud University Medical Center, Nijmegen, Netherlands; ^3^Social, Cognitive and Affective Neuroscience Unit, Department of Cognition, Emotion, and Methods in Psychology, University of Vienna, Vienna, Austria; ^4^Berlin School of Mind and Brain, Department of Philosophy, Humboldt-Universität zu Berlin, Berlin, Germany; ^5^Faculty of Philosophy, Philosophy of Science and Religious Studies, Ludwig-Maximilians-Universität München, Munich, Germany; ^6^Konrad Lorenz Institute for Evolution and Cognition Research, Klosterneuburg, Austria; ^7^Department of Psychiatry and Psychotherapy, Medical University of Vienna, Vienna, Austria; ^8^Medical Neuroscience Cluster, Medical University of Vienna, Vienna, Austria; ^9^Zukunftskolleg/Philosophy Department, University of Konstanz, Konstanz, Germany; ^10^Department of Neurology, Medical University of Vienna, Vienna, Austria; ^11^Department of Cognition, Emotion, and Methods in Psychology, Faculty of Psychology, University of Vienna, Vienna, Austria

**Keywords:** repetitive stereotyped behavior, basal ganglia disorders, predictive processing, active inference, aesthetic learning, art therapy

## Abstract

Recurrent, unvarying, and seemingly purposeless patterns of action and cognition are part of normal development, but also feature prominently in several neuropsychiatric conditions. Repetitive stereotyped behaviors (RSBs) can be viewed as exaggerated forms of learned habits and frequently correlate with alterations in motor, limbic, and associative basal ganglia circuits. However, it is still unclear how altered basal ganglia feedback signals actually relate to the phenomenological variability of RSBs. Why do behaviorally overlapping phenomena sometimes require different treatment approaches−for example, sensory shielding strategies versus exposure therapy for autism and obsessive-compulsive disorder, respectively? Certain clues may be found in recent models of basal ganglia function that extend well beyond action selection and motivational control, and have implications for sensorimotor integration, prediction, learning under uncertainty, as well as aesthetic learning. In this paper, we systematically compare three exemplary conditions with basal ganglia involvement, obsessive-compulsive disorder, Parkinson’s disease, and autism spectrum conditions, to gain a new understanding of RSBs. We integrate clinical observations and neuroanatomical and neurophysiological alterations with accounts employing the predictive processing framework. Based on this review, we suggest that basal ganglia feedback plays a central role in preconditioning cortical networks to anticipate self-generated, movement-related perception. In this way, basal ganglia feedback appears ideally situated to adjust the salience of sensory signals through precision weighting of (external) new sensory information, relative to the precision of (internal) predictions based on prior generated models. Accordingly, behavioral policies may preferentially rely on new data versus existing knowledge, in a spectrum spanning between novelty and stability. RSBs may then represent compensatory or reactive responses, respectively, at the opposite ends of this spectrum. This view places an important role of aesthetic learning on basal ganglia feedback, may account for observed changes in creativity and aesthetic experience in basal ganglia disorders, is empirically testable, and may inform creative art therapies in conditions characterized by stereotyped behaviors.

## Introduction

Repetition is a fundamental aspect of learning in humans. Especially in early stages of life, repetitive behaviors are part of normal development. Such transient expressions are thought to calibrate the brain to master rich, volatile, and still highly uncertain environments (Langen et al., 2011; Horst, 2013). However, repetitive behaviors can also become aberrant, exceed in intensity, persist beyond an adaptive window in normal development, interfere with daily functioning, and are present in different neuropsychiatric conditions (Ridley, 1994; Graybiel and Canales, 2001; Lewis and Kim, 2009; Langen et al., 2011; Muehlmann and Lewis, 2012). Such “repetitive stereotyped behaviors” (RSBs) constitute a class of rhythmic, ritualistic, or rigid movements, thoughts, and occupations, differing in frequency, and involving one or a combination of motor, sensory, or body-related responses. These can be broadly classified between generally lower-level behaviors such as repetitive movements, manipulations of objects, or self-injury, and higher-level behaviors such as repetitive language, insistence on sameness, restricted interests, or complex rituals such as behavioral routines, counting, sorting, or hoarding (Ridley, 1994; Graybiel and Canales, 2001; Watt et al., 2008; Lewis and Kim, 2009; Langen et al., 2011; Muehlmann and Lewis, 2012).

Many RSB-related symptoms are, in turn, diagnostic hallmarks of obsessive-compulsive disorder (OCD) and autism spectrum conditions (ASC). OCD is a disabling mental disorder involving obsessions (repetitive and intrusive thoughts, urges or images) and compulsions (repeatedly performing certain behavioral or mental routines) (Stein et al., 2019). ASC is a neurodevelopmental condition characterized by repetitive behaviors, difficulties in social interaction and communication, and atypical perceptual processing (Sharma et al., 2018). RSBs also manifest in a number of patients with neurodegenerative disorders such as Parkinson’s disease (PD) (Ridley, 1994; Alegret et al., 2001; Graybiel and Canales, 2001; Lewis and Kim, 2009; Langen et al., 2011; Muehlmann and Lewis, 2012), especially in phenomena such as punding—an intense focus on seemingly pointless, repetitive activities and manipulations in long-term treated PD patients first recognized in stimulant overuse (Evans et al., 2004; O’Sullivan et al., 2007; Spencer et al., 2011). As such, due to the role of RSBs as a major class of symptoms obstructing individuals’ lives, and even as a core neurobiological, cognitive, or physical fundament of brain conditions, research demands that the neurobiological basis of RSBs be better explained.

However, precisely because of their heterogeneity, especially within individual disorders, and precluding a purely phenomenological classification, RSBs are insufficiently understood. There is no clear mapping between underlying neurobiological substrates and observable repetitive phenomena (Yerys, 2015). Aside from distinct lesions or interventions, correlations between brain structure and complex mental health phenotypes may be small (Marek et al., 2022). Additionally, from a network dynamics perspective, similar neurobiological alterations may give rise to differing phenomena, and conversely, similar phenotypical expressions may rest on differing causal factors (Durstewitz et al., 2021). This hints at a need for a broader conceptual basis to explain RSBs.

Interestingly, such an answer might be found by comparing across several disorders associated with RSBs, by looking to emerging evidence for specific shared, or in cases, differentiated functioning of specific basal ganglia structures modulated by dopamine, and by framing our assessment along emerging theory regarding predictive processing and aesthetic learning (see below).

A frequent finding in the various conditions that show RSBs are alterations in basal ganglia structures and sensitivity to changes of the tone of dopamine—one of the main neuromodulators in the brain and particularly in the basal ganglia (Canales and Graybiel, 2000; Graybiel et al., 2000; Langen et al., 2011; Averbeck et al., 2014; Maltête, 2016; Katherine, 2018). Accordingly, a neurobiological stratification for RSBs has been suggested along the affected parallel functional loops of the basal ganglia with the tripartite model, i.e., sensorimotor, associative, and limbic circuits (Langen et al., 2011; Yerys, 2015). Going beyond a purely anatomical correlation, the ubiquitous feedback function provided by the basal ganglia loop to all cortical areas needs to be taken into account. At this point, the cognitive framework of predictive processing promises new insight, since it has been successfully employed to describe several conditions with basal ganglia involvement in apparently dichotomous ways, which we will demonstrate here by comparing ASC, OCD, and PD. Predictive processing describes the brain as a fundamentally predictive organ that attempts to model its inherently variable environment. Such predictive models are generated from, and constantly tested against, external sensory information in the service of reducing uncertain, surprising, and therefore potentially threatening encounters. Importantly, to resolve any mismatch between *expectations* and *observations*, the brain can place preferential weight on either one over the other, thereby profoundly altering the process of sense-making about the surrounding world. Furthermore, this may become visible in preferential ways of creative expression and communication. We will therefore explore to what extent basal ganglia feedback signals modulate the weight placed on new sensory signals, and propose that RSBs may be precipitated by unusually high or low levels of perceived uncertainty about action-outcome mappings.

Ultimately, looking through this predictive processing lens suggests a perception-related learning capacity of the basal ganglia that appears to be well described by aesthetic learning, which is the final concept to be introduced here. We use the term aesthetic learning in the sense of active experience based on “newly perceived things” in contrast to “things known” (Wickman, 2012), reflecting the common thread in this paper of an external-internal perceptual spectrum. Aesthetic experience is marked by specific affective values and relates to personal tastes and appreciation (Kant, 1790). Here, the concept of aesthetic experience falls under the more general concept of experience as meaning-making with respect to environmental sensory stimuli, and could be seen as a specific experience of learning (Dewey, 1934; Wickman, 2006). Aesthetic learning and the bringing forth of new meaning can be achieved by engagement, among others, with cultural artifacts (Fingerhut, 2021), artworks (considered as highly salient, attention-amplifying objects, see also Sarasso et al., 2020a,b), and also natural landscapes (Menatti and Casado da Rocha, 2016; Heras-Escribano and Pinedo-García, 2018). Aesthetic learning is associated with dopaminergic modulation of sensorimotor, associative, and limbic basal ganglia circuits (see also Spee et al., 2018) and appears to influence action-perception cycles and environmental adaptation (Sarasso et al., 2020a,b).

**FIGURE 1:**
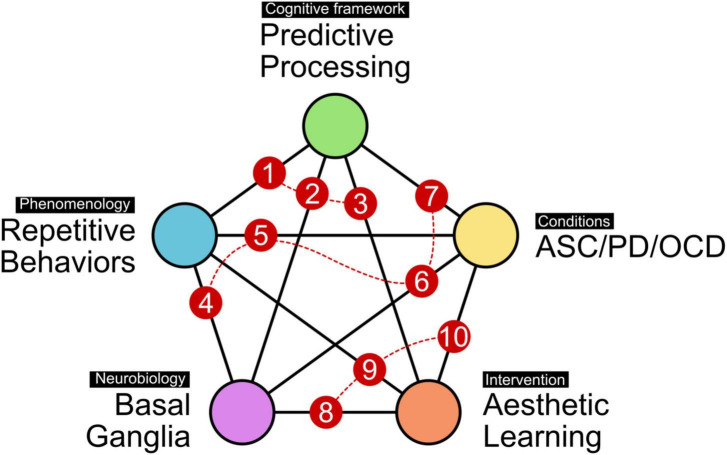
Thematic landscape for approaching RSBs. The present paper assumes five interconnected themes of relevance. These include the phenomenology itself (i.e., observable behavior), the basal ganglia as a likely neuroanatomical and neurophysiological substrate, the cognitive framework of predictive processing as a high-level model of brain function, exemplary conditions selected for this discussion (ASC, PD, and OCD) which are partly characterized by RSBs, and lastly, how aesthetic learning emerges from, and interacts with, this thematic space. The numbering of pairwise connections provides a guide through the present paper. Sections “Repetitive stereotyped behavior and predictive processing (1), Predictive processing and basal ganglia (2), and Predictive processing and aesthetic learning (3)” will discuss how predictive processing relates to repetitive behaviors, neurobiology, and aesthetic learning. Sections “Repetitive behaviors and basal ganglia function (4), Repetitive phenomena in autism spectrum conditions, obsessive-compulsive disorder, and Parkinson’s disease (5), Basal ganglia involvement in autism spectrum conditions, obsessive-compulsive disorder, and Parkinson’s disease (6), and Predictive processing accounts of autism spectrum conditions, obsessive-compulsive disorder, and Parkinson’s disease (7)” will discuss the phenomenologies of the exemplary conditions, and how they relate to neurobiology and to the cognitive framework of predictive processing. Lastly, sections “Aesthetic learning and basal ganglia function (8), Aesthetic learning in repetitive stereotyped behaviors (9), and A role for aesthetic learning in autism spectrum conditions, obsessive-compulsive disorder, and Parkinson’s disease (10)” will focus on how basal ganglia circuits might influence aesthetic experience, and what this could mean for art and occupational therapies in conditions showing RSBs. Specifically, directing sensory salience through strategic contexts and learning environments may help to counterbalance skewed weighting of feedback signals by the basal ganglia, providing individual space for action, experience, and communication. Suggested key literature on all pair-wise connections: [1] (Perrykkad and Hohwy, 2020) [2] (Friston K. J. et al., 2012; Shine, 2020) [3] (Van de Cruys and Wagemans, 2011b) [4] (Langen et al., 2011; Redgrave et al., 2011) [5] (Alegret et al., 2001; Stein et al., 2019; Crespi, 2021) [6] (Yerys, 2015; Elkouzi et al., 2019; Li et al., 2021) [7] (Friston K. J. et al., 2012; Pellicano and Burr, 2012; Lawson et al., 2014; Friston et al., 2015; Levy, 2018; Kiverstein et al., 2019) [8] (Pelowski et al., 2017; Spee et al., 2018) [9] (Ferrell, 2015) [10] (Kesner, 2014; Lhommée et al., 2014; Seth, 2019).

Equipped with this clinical, neurobiological and psychological background, our aim in this paper is to contribute to the understanding and management of RSBs with a structured discussion along the connections between the five broad concepts introduced here (to make the discussion easier to follow for the reader, Figure 1 provides a visual guide through the organization of this paper, together with key literature on the respective connections between concepts). To recapitulate, these include (i) the phenomenology of repetitive behaviors, including how they manifest in creative expression, (ii) their relation to the cortico-basal-ganglia system, (iii) the cognitive framework of predictive processing operating on the principle of uncertainty reduction, (iv) exemplary clinical conditions (ASC, OCD, PD) that are characterized by repetitive behaviors, show basal ganglia involvement, and can be described in predictive processing terms, and lastly, (v) aesthetic learning and how it may contribute to strategies and interventions suited to modulate RSBs in neuropsychiatric conditions. This means both ameliorating those behaviors that are experienced as negative or debilitating, and incorporating or accepting RSBs that may be compensatory or meaningful.

Based on this review, our overall proposal is that RSBs represent behavioral patterns in response to unusually high or low perceived levels of salience and uncertainty. This may result from external environmental circumstances or from alterations of the internal apparatus for generating probabilistic models of the environment, as posited by the predictive processing framework. We will argue that the gain function provided by the basal ganglia modulates not only movement vigor (Yttri and Dudman, 2018), but likely also the salience of sensory perceptions and action opportunities. Because salience, surprise and uncertainty are related in predictive processing (Friston, 2009; Friston K. J. et al., 2012; Parr and Friston, 2018), basal ganglia output signals should therefore directly influence perceived uncertainty. Salient features of the environment that are novel and/or surprising require recalibrating existing knowledge. Accordingly, gain computation by the basal ganglia may balance previous experience with new sensory information by shifting the relative weighting from existing prior knowledge to new external signals. In this light, we suggest that RSBs might manifest in two possible ways. Firstly, insufficient gain reduces the salience of external stimuli, and individuals are more likely to remain in established, prior learned, perseverative expectations and patterns of activity (exemplified by OCD). Secondly, excessive gain produces indiscriminate and overwhelming salience resulting in unsettlingly poor predictability, and individuals might engage in repetitive sensory sampling to reduce uncertainty, and/or chronically adapt their behavior to limit perceptual exposure through restricted interests (exemplified by ASC). Finally, we will propose that this framework has implications for occupational and art therapy in neuropsychiatric disorders characterized by RSBs. Specifically, aesthetic learning principles may help to adjust the relative weighting of external signals and internal predictions by guiding the salience and predictability of stimuli and contexts, and by increasing the tolerance for ambiguous stimuli (Sarasso et al., 2020a,b; Fingerhut, 2021).

## Repetitive stereotyped behavior and predictive processing

### “Normal” repetitive behavior

Repetitive behaviors can be functional as part of normal learning and development (Langen et al., 2011; Horst, 2013). Learning, under the umbrella of predictive processing, is a reciprocal process that involves multiple stages as well as loops of experience and behavior to be successful. Successful means that what is learned is also meaningful and purposeful to the context, person, and environment (Friston et al., 2016). One essential process to this successful adaptation is to match sensory information (exteroceptive, interoceptive, and proprioceptive) and prior knowledge (a collection of experiences stored as prior beliefs informing action policies, Friston et al., 2009). If the environment is not yet predictable, thus new and uncertain, there are several elements needed to collect information, starting with attention to the salient features, perception of these, and then evaluation of their relevance in a given context. Alongside these elements, behaviors are initiated as an informed guess about resulting perceptions. Repetition is a major strategy to test the relevance and efficacy of the behavior and to establish reliable action-perception mappings (environment-adapted meaningful behavior). That information can then be stored as a posterior belief for the future (Friston et al., 2016). In this way, relevant sensory information is embedded and integrated either within existing prior beliefs (re-evaluating) or serves to establish new beliefs (typical at young age).

Discernible categories for patterned repetitive behaviors that are not necessarily pathological include habits, tics, stereotypies, and fidgeting. Habits comprise learned, automatic sequences of action in well-known contexts that are more or less purposeful (Graybiel, 2008). Tics are semi-voluntary non-rhythmic and sudden motor or vocal expressions that relieve an irresistible inner urge (Martino and Hedderly, 2019). Stereotypies are repetitive patterned movements that might be experienced as soothing and enjoyable, may focus attention, and can be stopped by distraction (Péter et al., 2017). Fidgeting comprises seemingly purposeless activities such as rocking on a chair, clicking a pen, bouncing a leg, nail-biting, or playing with one’s hair, all of which are expressed more strongly during times of boredom or stress. Because fidgeting provides very precise action-outcome mappings, it might support self-evidencing and a sense of control in both under- and overwhelming environments (Perrykkad and Hohwy, 2020).

Another related phenomenon is superstitious behavior, or rituals for changing one’s luck. These can have repetitive character and may give a false sense of causality in situations of uncertain or inexistent action-outcome relations. This was famously demonstrated in the classical pigeon conditioning experiments by Skinner (1948). A possibly corresponding phenomenon has been termed “repetition bias,” describing the experimental observation that phasic dopamine release evoked by sensory stimuli reinforces the immediately preceding behavior, and that high dopamine levels cause behavioral repetition (Redgrave et al., 2011). Stereotyped behaviors may also appear in timekeeping: animals improve performance in interval-timing experiments by generating repetitive activities, and humans do the same (Buzsáki, 2019, 260).

In the following, we will describe how repetitive behavior as a learning strategy is considered in the current predictive processing framework of brain function, which especially focuses on how humans constantly generate and update beliefs about the environment by comparing them to sensory information.

### Predictive processing

Predictive processing is a rapidly emerging framework in cognitive science and theoretical neuroscience (Friston, 2010; Hohwy, 2013; Clark, 2015; Wiese and Metzinger, 2017). This far-reaching approach describes the mind as an embodied probabilistic model of the world that constantly makes predictions about the causes of its sensory states (Hohwy, 2013; Clark, 2015). Mechanistically, predictive processing posits that the brain attempts to minimize any mismatch between predicted and actual sensory information (i.e., prediction error, or surprise) by continuously and iteratively updating hierarchical generative models of the hidden statistical regularities of uncertain and volatile environments acting upon inherently noisy sensory systems. Within the multi-level hierarchical architecture of the brain, the predicted “virtual version of the sensory data” (Clark, 2015, 25) streams downward and is compared to a flow of upstreaming signals informing higher-order cortical regions. These upstreaming signals are the residuals that have not yet been explained away at cortical levels of lower abstraction.

The prediction of activity at lower cortical areas by higher cortical areas has been demonstrated for example in the visual system, and this perceptual implementation falls under the term of predictive coding (Hohwy, 2013; Spratling, 2016). However, perception is an inherently active process, because the testing of predictions against sensory information involves the targeted perturbation of the observable environment through movements and actions, and this is emphasized by the active inference account (Friston K. et al., 2017; Pezzulo et al., 2018; Parr et al., 2022). Empirically, perception is strongly facilitated by movement, be it through microsaccades of the eyes, moving the fingers across a surface, rhythmic sniffing, or turning toward the source of a sound. Here, the selection of behavioral policies is influenced by prior expectations such that actions are most likely to lead to sensory information that matches prior expectations or resolves uncertainty. The active inference account places an emphasis on the operational role of cognition (Friston K. et al., 2017; Pezzulo et al., 2018; Parr et al., 2022). Within this framework, the primary function of cognition is not a passive encoding of the world but to allow for maintaining the organism’s self-organization in the context of an ever-changing environment. Accordingly, cognition plays a role in self-regulation in response to metabolic demands. This process occurs not in solipsistic isolation but within a dynamic world that is not only a physical environment but also inhabited by other cognitive agents. This view on predictive processing leads to two important consequences: *preferences* and *affordances*.

Priors and prediction errors encode preferred metabolic states and deviations from them. To not just passively endure these deviations, actions allow the agent to counteract them, i.e., to transition away from an undesired to a more preferrable state. For example, the perception of thirst can be seen as a mismatch between preferred hydration levels and the *status quo*. This prediction error can be decreased on a perceptual level, e.g., by habituation (i.e., getting used to being thirsty) or meta-cognitive strategies like distraction. Alternatively, the agent can act against this undesired state and seek out opportunities to consume a drink. The success of this behavior not only depends on the agent’s skills but also on opportunities provided by the environment. These action opportunities within an environment are commonly described by the concept of affordances (Gibson, 1978; Rietveld and Kiverstein, 2014). Affordances are not properties of the agent or environment alone, but emerge from their mutual interaction. In this way, the process of minimizing prediction errors or surprise allows organisms a sustained and homeostatic exchange with their environments, and is considered a mandatory requirement for any self-maintaining biological system and a possible unifying principle for understanding perception, action, attention, experience, and learning (Friston, 2010; Ramstead et al., 2018). Nonetheless, beyond homeostatic adaptation to ever-changing environments, humans in particular are also curious and seek surprise and novelty in non-threatening settings (Clark, 2018). Such information foraging is greatly facilitated by aesthetic objects and experiences (Van de Cruys, 2017).

### Repetitive behavior and uncertainty in predictive processing

In general, any discrepancy between model prediction and sensory signal could be dissolved in one of two ways: the first way is to rely on the sensory signal and update the model, and the second way is to insist on the model and try to change the world through action so that it matches expectations (Parr et al., 2022). This dichotomy is also present in Bayes’ Theorem, one of the basic mathematical foundations of predictive processing (Box 1).

**Box 1. The dichotomy of Bayes’ Theorem.**

**FIGURE 2:**
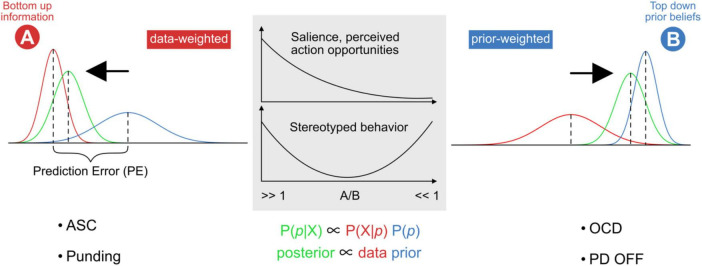
Model of externally versus internally weighted dynamics in predictive processing. The probability distribution and precision of posterior beliefs (posterior; green) are proportional to bottom-up sensory information (**A**; data; red) and top-down prior expectations (**B**; prior; blue). The output of the basal ganglia contributes to setting the relative weighting between data and priors, by influencing the relative gain between external (exteroceptive) and internal (predictive) signals. Stronger relative weighting on prior beliefs increases the resistance to switching away from the current state or trajectory. Stereotyped repetitive behaviors might appear at both far ends of this spectrum. Overfitting describes the situation where a model accounts for too much detail and random fluctuations at the expense of generalizability (“data-weighted”). An under-fitted model regularizes too much and does not adjust sufficiently for relevant deviation in the data (“prior-weighted”). ASC and PD-on phenomena such as punding can be placed on the left side, OCD and PD-OFF on the right. Further description in the text.

A basic premise of predictive processing is that the brain enacts a form of Bayesian inference (Gershman and Uchida, 2019; Isomura, 2021). Here, the posterior P(p| X), i.e., the updated probability of a belief state p given sensory information X, is proportional to two factors: (i) the expected likelihood of experiencing sensory states given prior beliefs P(X| p), and (ii) the probability of the current best guess P(p) about the hidden causes for sensory states, i.e., the current generative model. The relative precision between these two factors can be skewed toward one or the other, such that ensuing prediction errors are more or less likely to update prior beliefs (Hohwy, 2013) (see also Figure 2).

The picture gets more complicated though, because expectations about sensory signals consist of two parts, namely the expected signal magnitude, and the expected precision, akin to the size of an effect and its standard deviation (Lawson et al., 2014; Clark, 2018; Ransom et al., 2020). If a sensory signal is expected to be very reliable, it will be weighted higher relative to the existing model, likely through some form of a neural gain mechanism. Note that while passing through ascending cortical hierarchies, sensory signals and prediction errors can be treated as practically identical−both are bottom-up, and both are subject to top-down precision expectations (Ransom et al., 2020). As discussed further below, we assume that throughout cortical hierarchies, basal ganglia feedback is an ideal candidate for providing such a gain signal (Shine, 2020). The point to emphasize here, is that higher gain on prediction error signals also means that ongoing experiences appear rather surprising, novel, and salient (both on the exteroceptive, sensory input side as well as on the proprioceptive motor side). Such increased precision expectations therefore cause uncertainty, since existing prior models apparently are not reliable enough to explain away a myriad of surprising signals. Accounting for excessively salient details also results in overfitting and poor model generalizability. Intuitively, repetitive behavior should serve to increase model reliability, while prediction errors and surprise should continuously diminish from one iteration to the next. This means that repetitive behavior might represent a compensatory strategy in response to abnormally high precision expectations about upcoming sensory signals and/or low reliability of internal models. Furthermore, an individual could attempt to reduce unsettling levels of uncertainty by actively restricting behaviors such that sensory exposure is limited to narrow, recurrent, and predictable windows into the world. This scenario fits well with predictive processing accounts of ASC (Palmer et al., 2017) and with the phenomenon of punding in PD.

In contrast, gain on sensory signals may be too low, rendering existing prior models relatively overweighted. Models then represent strong attractor states that resist relaxation and updating, even when confronted with strong conflicting sensory evidence. This scenario seems to fit, e.g., with perseveration despite changed circumstances, and has been proposed to underlie certain OCD symptoms (Levy, 2018).

**FIGURE 3:**
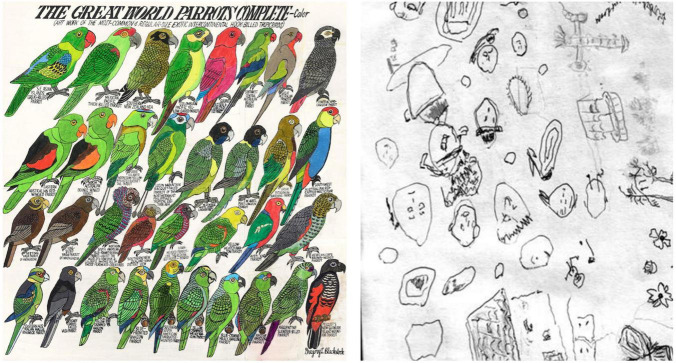
**(Left)** Drawing by the Seattle artist Gregory Blackstock (Blackstock, 2006), whose extraordinary visual lists, while unique in their style, seem to convey a distinctive autistic cognitive mode (see also discussion in Roth, 2020) (reproduced with kind permission from the artist; courtesy Greg Kucera Gallery). **(Right)** Drawings of a PD patient with punding (Reproduced from O’Sullivan et al., 2007 with permission from BMJ Publishing Group Ltd.). Notice the creative use of visual likeness, repetition, and systematic modulation of objects. Small, repeating themes with slight variations suggest an apparent lack of generalization and overfitting to (random) variability, with many equally salient details, all seemingly deserving a category of their own (hyperspecific perceptual categories). Parallels may also exist with a published case report describing a graphic designer suffering from PD who obsessively created numerous variations of specific themes (Chatterjee et al., 2006; reviewed in Lauring et al., 2019a). Contrast the previous examples with the order, purity, regularity, repetition and control in the work of Yayoi Kusama (Ferrell, 2015), who expresses her experience of OCD, often employing polka dots, oblong organic shapes and infinity mirrors, with a conspicuous absence of systematic variation of details. Rather, these shapes represent grand categories with extensive symbolic weight, repeated ad infinitum (Ferrell, 2015) (Yayoi Kusama: “The Spirits of the Pumpkins Descended into the Heavens” (2017), National Gallery of Australia, Canberra, Australia; https://en.wikipedia.org/wiki/Yayoi_Kusama#/media/File:Kusama_Yayoi_The_Spirits_of_the_Pumpkins_Descended_into_the_Heavens.jpg). Further discussion in the text.

**FIGURE 4:**
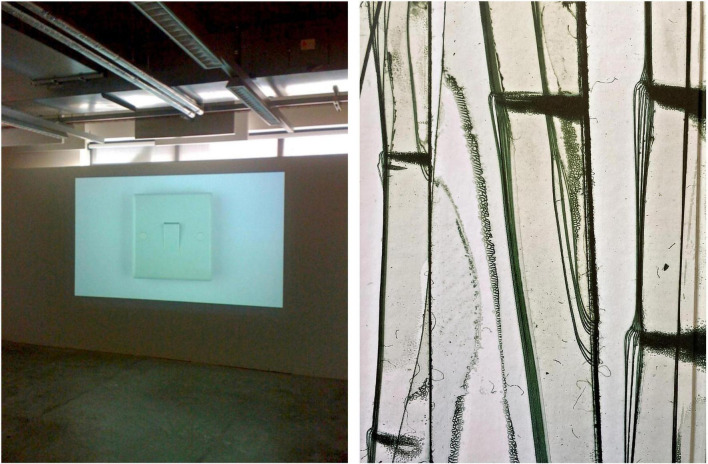
Two exemplary artworks that can be interpreted as revealing contrasting top-down and bottom-up perceptual modes. **(Left)** “Obsession, Compulsion, and the Switch,” installation (2010), discussed in a Ph.D thesis titled “An Artistic Equivalence of my Obsessive Compulsive Disorder” (Baugh, 2015). A film of a light switch was projected onto a wall and played on a loop, alternating between bright light and darkness, with the intention to replicate the experience of repeatedly turning the switch off and on. This artwork implements themes of control and repetition, and features the image of a symmetrical, prototypical, predictable, and almost flawless object (reproduced with permission). **(Right)** “Scotch Tape,” color photo, 20 × 30 cm (2004), by an artist affected by advanced PD. The artist has been “meticulously documenting changes within her immediate urban and rural environment,” she “collects and classifies, assembles and arranges, prepares and analyses […] the unseen everyday world, […] to pay homage to the beauty of the banal.” Symmetry is of no relevance in her work, in which she reveals otherwise overlooked structure and variable patterns in the detail. Although these are selective examples, it is tempting to speculate that for an individual with OCD, similar levels of detail might be perceived as contamination. Reproduced with permission, © Brigitte Gauss, Zeitstrukturen, Kaleidoskop der unbesehenen Alltäglichkeit, Hrsg Edith Almhofer, DEA Buch- und Kunstverlag, Wien, 2004.

In short, if the gain on sensory signals is too high (and/or if existing prior models are relatively underweighted), it *may help* to keep doing the same thing over and over again. If the gain on sensory signals is too low (and/or if existing prior models are unusually overweighted), the system *can’t help* but to keep doing the same thing over and over again. Curiously, while repetitive behaviors are present in both ASC and OCD, there appear to be subtle differences that can be appreciated in patterns of creative expression, as shown in Figures 3, 4.

## Predictive processing and basal ganglia

In clinical neuroscience, predictive processing offers an elegant link between psychology and biology at the mesoscale of neuronal networks and has been applied to various neuropsychiatric conditions (Smith et al., 2021). Although the predictive processing framework is neuroanatomically plausible, the exact structural implementation in specific neural circuits is still debated (Bastos et al., 2012; Keller and Mrsic-Flogel, 2018; Shine, 2020; Isomura, 2021). While several accounts focus primarily on the neocortex (Bastos et al., 2012; Keller and Mrsic-Flogel, 2018), it has been pointed out that subcortical structures, in particular basal ganglia and cerebellar feedback loops, would be suited to balance the relative weighting of prior beliefs and prediction errors (Shine, 2020). The basal ganglia are a group of subcortical nuclei forming the cortico-striato-thalamo-cortical feedback circuit, but also connect to brainstem and other structures in the central nervous system (Bergman, 2021). The feedback output from the basal ganglia has a strong inhibitory grip on thalamocortical connections, and this inhibition can be selectively lifted for specific signals that are temporally and spatially amplified. These permissive signals are typically associated with voluntary motor control, but are also involved in the learning of movements, procedural learning, habit and conditional learning, executive functioning, and emotion (Graybiel, 2005; Foerde and Shohamy, 2011; Seger and Spiering, 2011). According to the agency hypothesis, one of the implications of a system that selectively amplifies specific signals as a generic selector, is an ability to identify those external events that are reliably caused by own action (Redgrave et al., 2011; Bednark and Franz, 2014). This suggests that precise predictions are necessary for a sense of agency.

One of the key neuromodulators in this feedback loop is dopamine. Dopamine plays a central role in reward learning and motivation to learn (Gershman and Uchida, 2019), as well as in movement vigor, which can be seen most strikingly in PD: the specific loss of dopamine producing cells in the ventral tier of the substantia nigra is linked to the typical motor symptoms of the disease, particularly bradykinesia (Schapira et al., 2017; Humphries et al., 2018; Yttri and Dudman, 2018). The basal ganglia are topographically ordered in parallel loops that originate from, and project back to, the prefrontal, premotor, and motor cortex, but also to the sensorimotor and parietal cortex (Redgrave et al., 2010; Bednark et al., 2013) (see Box 2 for further discussion).

**Box 2. A very short primer on basal ganglia anatomy.**

The input layer of the basal ganglia consists of the striatum and subthalamic nucleus, and receives signals from deep-layer cortical neurons in the hyperdirect, direct and indirect pathways. These three pathways are active in temporal succession of net inhibitory (hyperdirect), net excitatory (direct), and again net inhibitory (indirect) action. Parallel loops also exert lateral inhibition. In this fashion, the basal ganglia provide temporally and spatially selective release of the inhibitory control exerted on the thalamus by the basal ganglia output nuclei (Itakura, 2015, 5; Wichmann and DeLong, 2016; Bergman, 2021). The feedback through the basal ganglia loop is strongly influenced by dopamine release in striatum and nucleus accumbens, from axons of dopaminergic cells in the substantia nigra pars compacta and ventral tegmental area. Dopamine has an overall permissive role, by activating excitatory dopamine D1 receptors of the faciliatory direct pathway, and by inhibitory dopamine D2 receptors on the indirect pathway.

It has become increasingly clear that motor and sensory processing cannot be separated but are deeply intertwined. Beyond learning, motor control, and selection of action plans, the basal ganglia loop has also been proposed to bias perception via the selection of distributed cortical “emulations” that include motor and sensory cortical networks (Colder, 2015). These emulations were proposed to contain representations of potential actions together with their associated expected perceptual consequences. In this way, the basal ganglia could prime the system for expected perception related to specific actions (Colder, 2015). This would closely link the basal ganglia to active inference models of action-guided perception (Friston K. et al., 2017). The emerging picture is that of a tight association, and even overlap, between action plans and expected sensory states, including proprioceptive predictions (Friston, 2011).

**FIGURE 5:**
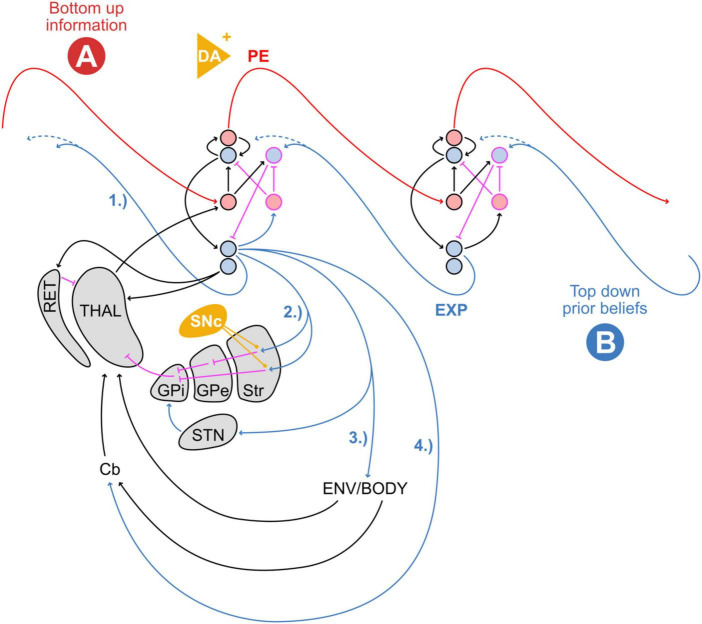
Schema of hierarchical cortical connections depicting several types of top-down prior expectations (EXP) from deep layer pyramidal neurons: (1) to lower cortical levels, (2) to the basal ganglia loops, (3) to the body and environment via the pyramidal tract, and (4) to the cerebellum in the form of an efference copy. The feedback from both the body and the environment arrives at the thalamus, where it is weighted by and integrated with basal ganglia signals at the level of the thalamocortical system. Striatal dopamine (orange ovoid) facilitates basal ganglia output and increases the precision of prediction errors (Friston K. J. et al., 2012). Cortical dopamine (orange triangle) modulates presynaptic input strength by influencing Hebbian plasticity (Isomura, 2021). Red: feed-forward, blue: feedback, black: other excitatory, pink: inhibitory connections, orange: dopaminergic modulation. DA, dopamine; PE, prediction error; EXP, expectation; RET, thalamic reticular nucleus; THAL, thalamus; GPi, internal globus pallidus; GPe, external globus pallidus; Str, striatum; STN, subthalamic nucleus; Cb, cerebellum. Cortical microcircuit adapted from Bastos et al. (2012). Different thalamocortical afferents from the thalamic core and matrix cells are omitted for clarity (Shine, 2020). The hierarchical cortical connection scheme is adapted from Bastos et al. (2012).

Figure 5 combines the classic cortical model of predictive processing (Bastos et al., 2012) with internal and external feedback loops through the basal ganglia and external environment, respectively. Here, top-down prior expectations result in prediction signals through several distinct, internal and external feedback loops relative to the neocortex. In particular, deep-layer cortical pyramidal neurons (DLPN) can be thought of as distributing prior expectations through various feedback loops: they project to lower cortical levels (1), to the basal ganglia loop (2), to the cerebellum via pontine nuclei (4), and in the form of the pyramidal tract (3) from the primary motor cortex to the muscles in the body that actively shape the temporal sequence of upcoming states of the body and sensory experiences. From this view, one can imagine primary motor cortical layer 5 Betz cells, that give rise to the pyramidal tract, not as simply sending motor commands. Rather, they would continuously propagate predictions of (i) upcoming proprioceptive signals expected from muscle spindles and Golgi tendon organs that signal physical body movements and positions, as well as (ii) exteroceptive signals through other sensory modalities. The ensuing prediction errors between predicted and current movement/position (as well as other sensations) are then iteratively eliminated through muscle action until expectations are matched.

At the level of the thalamocortical connections, the feedback from signals originating in the pyramidal tract is then processed differentially in core and matrix type thalamic neurons. Inputs through exteroceptive modalities, as well as signals from the cerebellum, including proprioception, arrive at core-type thalamic neurons which relay back to basal dendrites of DLPNs (Shine, 2020). In contrast, basal ganglia feedback tends to be parsed through matrix-type thalamic neurons which preferentially innervate and depolarize apical dendrites of DLPNs in a more widespread manner (Shine, 2020). When both types of input coincide, DLPNs can go into burst-firing mode. The co-occurrence of these two types of thalamocortical projections is suited to shape an unfolding dynamical landscape of sequential network states and trajectories (Shine, 2020). This seems to support the notion that the basal ganglia widen the landscape of affordances—possible future states and trajectories to select from Friston K. J. et al. (2012). In contrast, well-trodden habitual paths on that landscape are supported by the cerebellum, which enacts fast sequences of expected states in reliable temporal succession based on previous experience. According to this view, the basal ganglia might accomplish two seemingly separate functions. Firstly, they can positively or negatively reinforce ongoing activity and behavior (Yttri and Dudman, 2018), and secondly, they can broaden the range of possible actions by reducing the barrier for switching between them (Shine, 2020).

### Predictive processing and dopamine

According to predictive processing, the brain constantly infers states of the external environment, preferences about future states as well as policies, i.e., parameters for action sequences (Isomura, 2021). All of these components (states, values, and policies) are associated with changing levels of uncertainty due to inherently noisy and ambiguous observations (Gershman and Uchida, 2019). Dopamine relates in different ways to the precision of these components (Friston K. J. et al., 2012; Gershman and Uchida, 2019). It has been suggested to play a modulatory role by adjusting the weighting of different forms of prediction errors, including reward prediction errors, but also the weighting of salient sensory-motor cues that signify affordance (Friston K. J. et al., 2012; FitzGerald et al., 2015; Schwartenbeck et al., 2015; Gershman, 2017; Gardner et al., 2018). Striatal dopamine in particular plays a central role in encoding policy precision (Pezzulo et al., 2018; Gershman and Uchida, 2019), and through its modulation of basal ganglia output, it should also influence expected sensory information (Colder, 2015). Phasic dopamine is a teaching signal for instrumental or reinforcement learning (Redgrave et al., 2010). Interestingly, there are two kinds of instrumental control that could be seen as reflecting a weighting between more environment-driven versus more agent-driven dynamics. The first type concerns a reinforcement between environmental stimuli and subsequent agentic responses (environment-driven, stimulus-response). The second type of reinforcement concerns an action-outcome relationship (agent-driven, goal-directed) (Redgrave et al., 2010). These two forms of instrumental control were shown to be anatomically separated into dorsolateral sensorimotor regions (for automatic control) and rostromedial associative regions (for goal-directed control). The former is preferentially impaired in PD, which led to the suggestion that these individuals have difficulty in executing previously learned automatic sequences (Redgrave et al., 2010).

Within the predictive processing framework, striatal dopamine, by influencing basal ganglia feedback to the cortex, could be seen as a weighting mechanism that increases the likelihood of activating (sequences of) distributed cortical activity patterns together with their expected sensations (Colder, 2015), as well as facilitating potential state changes of the body-environment system (Shine, 2020). Because a lack of striatal dopamine would impair the facilitation of expected cortical sensorimotor activity patterns, this would blur the perceptual consequences of movements and actions, putatively biasing the system toward a default state or no change. This reflects the close association between motor and sensory systems seen, e.g., in PD (Patel et al., 2014). It also fits with the notion that striatal dopamine increases random exploration (Gershman and Uchida, 2019). Conceivably, changes in the propensity for trial-and-error exploration and novelty-seeking could be related to changes in artistic and creative expression before and after diagnosis and starting dopamine replacement therapy in individuals with PD (Bednark et al., 2016; Lauring et al., 2019a).

In summary, we suggest that the balance between prior models and incoming sensory data is dynamically modulated by basal ganglia output to distributed cortical networks. Basal ganglia output preconditions these networks to facilitate a selection between alternate potential futures. From an initial trial-and-error process, this eventually shifts to a sort of self-fulfilling prophesying, about sequenced combinations of states that the body will be perceived to be in, and about which states of the world will likely occur. Here, a spectrum unfolds between two polar opposites. On the one hand, overly precise existing models and prior expectations imply a relative insensitivity for current experiences. On the other hand, fuzzy existing models and/or overweighted prediction errors (new sensory information) could result in overfitting and widely salient and indiscriminate sensory-motor cues. With respect to (openness to) aesthetic experience, this account may help to understand the curious finding that art interest and creative expression change over the disease course in PD (Carson, 2014; Boot et al., 2017; Lauring et al., 2019a,b; Pelowski et al., 2020; Perez Matos et al., 2021), and may provide a mechanistic link between dopamine and creativity (Drago et al., 2009; Lhommée et al., 2014; Garcia-Ruiz et al., 2019), cognitive flexibility (Klanker et al., 2013), the association with explorative personality types (Deyoung, 2013), the evolutionary history of dopamine in arbitrating between exploitation and exploration (Cisek, 2019), as well as favoring pro-social and externally driven behavior (Yamaguchi et al., 2015; Raghanti et al., 2018).

## Predictive processing and aesthetic learning

Recently, researchers working in the field of predictive processing have addressed the arts and, either directly or indirectly, aesthetic learning (Van de Cruys and Wagemans, 2011b; Kesner, 2014; Friston K. J. et al., 2017; Van de Cruys, 2017). A major focus of these papers is discussing “why” we engage with stimuli that potentially violate our predictions, such as artworks, along with aspects of learning, and as counterpoint to a debate of a preferential “dark room scenario” (Clark, 2013; Froese and Ikegami, 2013; Little and Sommer, 2013). The idea behind the latter is that an agent seeking to minimize prediction error would prefer a dark room because prediction errors can be maximally reduced within such a space. However, a dark room, from the perspective of predictive processing, does not correspond to the expected world (at least not permanently, Friston K. et al., 2012) and based on the experience within a social-cultural space. Paradoxically, a dark space would then be an unexpected event, whereas some degree of surprise is an expected event.

Within a hierarchical architecture, the goal is to make violations of prediction error reduction predictable, by expecting surprise within one level and expecting potential epistemic gain on another. Such error rates can be used as a learning signal (including rate of epistemic gain) to increase optimal adaptation in an ever-changing world (Van de Cruys, 2017). An enculturated mind (Fingerhut, 2020), thus, anticipates an enhanced learning effect in certain spaces (such as museums, unfamiliar landscapes, cultural spaces, etc.) or with certain objects considering the expectation of surprise (potentially stored within habits as meta-model and habitual policies), but with the value of increased information gain.

This interlude (expectation of surprise at a meta-level in favor of increased prediction reliability) is particularly utilized by artists who violate carefully established viewer predictions. However, sometimes the viewer can recreate the predictability within the broken pattern by allowing a heightened sensory information gain, associated with intensified affect and aesthetic value. This also seems to be at the core of so-called aesthetic emotions, often connected to dopamine (Spee et al., 2018), that can amplify perception and engagement (Fingerhut and Prinz, 2018; Sarasso et al., 2020b).

That said, cognitive models not only influence how we perceive the world but also how we interact and engage with artifacts (including artworks) and different media in specific contexts (Fingerhut, 2020, 2021). The context surrounding aesthetic stimuli, which might be displayed in a museum (see also for further reading the white cube phenomena; O’Doherty, 1986) or graffiti on the street, is relevant for establishing an aesthetic learning atmosphere. This means that not only are aesthetic objects salient objects (objects with intriguing affordance due to their epistemic value potential), but the atmosphere surrounding these objects is part of meta-beliefs considering socio-cultural behavior, hereby allowing a person to “get into”—on a cognitive and affective level—aesthetic learning processes. Here we would also situate aesthetic learning: namely related to the experience and the reward that people derive from training their senses and gaining new insights mostly about their socio-cultural environment but also about other personal viewpoints, emotional experiences, ideals, object presentation, or variety of object usage.

In short, humans appear as if they are actively seeking out prediction errors through trial-and-error exploration or experience-guided information foraging. This process can feel rewarding (affective value) for two reasons. Firstly, due to the empirical information gain (such as being surprised about a hidden meaning of an artifact, gaining new perspectives), and secondly, due to instrumental self-evidencing that challenges habitual experience (e.g., when someone is aware of his/her own perception and feels excitement about novel insight). Both aspects enable counterfactual models of the world (for example, a blue tree), which enhance the experience of reality but also the tolerance for, and even pleasure of, ambiguity. In addition, through affective value and cognitive enrichment, aesthetic experiences can even grasp deep into humans’ existential values (see for example sublime or transformational experiences, Pelowski, 2015; Pelowski et al., 2021). Beyond personal epistemic value, aesthetic learning shapes social and cultural interactions (Kesner, 2014; Friston K. J. et al., 2017). One reason is that common artifacts induce similar patterns of meaning, emotion, opinions, and predictions in social groups. Art and imagery as a medium of intergroup communication can create common understanding even when other communication mechanisms might fail, such as spoken language. Experiences with cultural aesthetic objects are even a key element of the anthropology and history of knowledge acquisition. This also directly addresses humans’ engagement with socio-cultural objects, as since the first cave-paintings, aesthetically appealing creations conceptualize efficient knowledge transfer considering current state of lived-in environments (Dissanayake, 2008, 2009).

**FIGURE 6:**
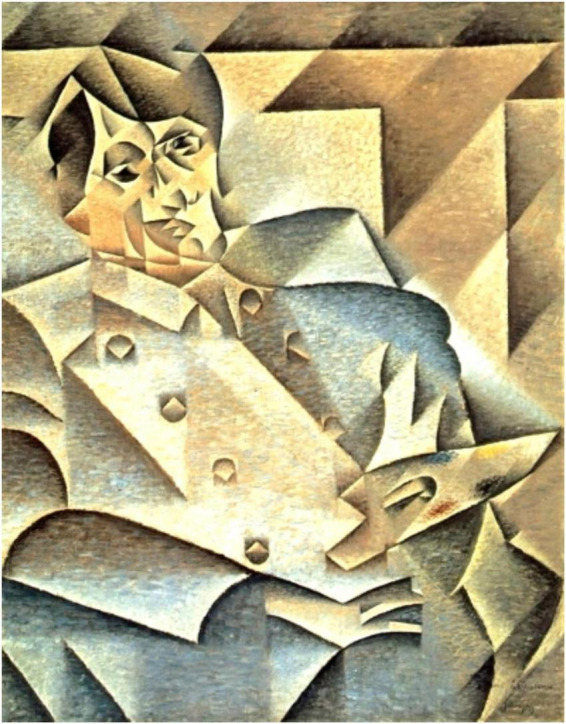
Cubistic image of artwork *Portrait of Pablo Picasso*, Juan Gris (1912) Copyright information: Shown works are in the public domain in its country of origin and other countries and areas where the copyright term is the author’s life plus 70 years or fewer. These works are in the public domain in the United States because it was published (or registered with the United States Copyright Office) before January 1, 1926 (for image search and copyrights: https://commons.wikimedia.org/wiki/File:JuanGris.Portrait_of_Picasso.jpg).

Additionally, artworks and art production can be seen as an intensified form of engagement that aims at challenging our every-day habits (or re-habituating us) in ways that makes them interesting to consider also for therapeutic applications. Considering the visual arts, for example, one way to increase salience and perceptual signaling is the amplification of low-level features and attributes in artwork. An example would be the usage of non-naturalistic colors or saturation, as found, e.g., in impressionistic styles. Another way to gain attention, or increase prediction error, is to distort and reassemble parts of an object from different perspectives. This has been done in diverse kinds of artworks that are more abstract, but most prominently visible in cubism (Van Geert and Wagemans, 2020). Figure 6 presents an example of cubism, *Portrait of Pablo Picasso*, Juan Gris, 1912, showing the re-assembling of diverse perspectives of face elements (eyes, nose, and mouth). Despite the abstractness, humans are usually able to detect a person despite the experienced ambiguity and unrealistic new compilation of body parts by using their prior models. In the process, they may also update their view of how a thing, object, or person can be depicted out of different perspectives. This update supports reducing uncertainty in the future, learning about, e.g., diversity in perspective, color usage, etcetera. The argumentation for humans’ affection to unpredictable and ambiguous stimuli, if they happen within a safe space, has already been suggested in pioneering work (Van de Cruys and Wagemans, 2011b; Clark, 2013, 2015; Schwartenbeck et al., 2013; Kesner, 2014; Friston K. J. et al., 2017; Seth, 2019; Sarasso et al., 2020b). To summarize, the researchers herein debate that although the predictive processing approach ultimately argues that our predictive mind aims to maximize predictability or minimize prediction errors (Friston, 2010), the neural networks within our cognitive system seem to require a certain amount of unpredictable stimulation to adapt to the constant fluctuations within the environment (Clark, 2015, 2018). In other words, a fully predictable engagement with the world without any surprises or prediction error would feel unpleasant. It would violate our expectation that the world is constantly fluctuating and should provide us with constant opportunities for experiencing novelty and learning.

## Repetitive behaviors and basal ganglia function

Although stereotyped behavioral expressions are sometimes adaptive, they can also become pathologic and maladaptive, can severely disrupt daily functioning, may become socially detrimental, or may even involve self-harm. Detrimental RSBs are mostly observed along specific conditions and described in the context of their respective clinical picture. Therefore, potential common underlying brain network abnormalities are more difficult to detect (Langen et al., 2011). Nonetheless, associations with underlying structural and functional brain abnormalities have been described (Lewis and Kim, 2009; Muehlmann and Lewis, 2012; Gao and Singer, 2013; Francis et al., 2014; Péter et al., 2017; Martino and Hedderly, 2019; Keller et al., 2021; Ganos et al., 2022), most consistently an involvement of basal ganglia structures and sensitivity to changes in dopaminergic tone. For example, motor stereotypies can be induced pharmacologically in animal models by dopamine receptor agonists or drugs that increase synaptic dopamine (Péter et al., 2017), and vascular lesions in different basal ganglia structures can provoke repetitive phenomena (Benke et al., 2000; Langen et al., 2011; Péter et al., 2017; Ganos et al., 2022). Because parallel basal ganglia loops are connected in similar fashion to all cortical areas (Redgrave et al., 2010), the computations they perform should be largely analogous, along the lines of a generic selection and reinforcement process (Redgrave et al., 2011). A common grouping of the parallel basal ganglia loops, with fluid transitions, differentiates limbic, associative, and sensorimotor circuits. These circuits are assigned with motivational, cognitive, and motor control, respectively (Redgrave et al., 2010; Langen et al., 2011). To some extent, different repetitive phenomena follow the functional connectivity of the circuits in this tripartite model (Langen et al., 2011; Yerys, 2015). For example, in ASC, the limbic loop has been implicated with hyperresponsiveness to non-social stimuli and circumscribed interests, the associative loop with impaired top-down behavioral control and perseverative behavior, and the sensorimotor loop with stereotypic movements. However, the mapping between phenomenology and alterations in structural and functional magnetic resonance imaging studies is incomplete (Yerys, 2015). Aside from basal ganglia involvement in ASC, OCD, and PD, many other conditions display RSBs linked to basal ganglia and dopaminergic abnormalities as well, including Tourette syndrome, Huntington’s disease, addiction, attention deficit hyperactivity disorder, and postencephalitic and vascular basal ganglia lesions (Benke et al., 2000; Langen et al., 2011).

Importantly, not only neurobiological factors, but also external, contextual factors influence RSBs, including impoverished environments or restricted social interactions. For example, primates reared in isolation, or reared by peers instead of the mother, show increased stereotypies (Lutz, 2014). Similar observations were made for children raised in orphanages (Péter et al., 2017). Furthermore, animals may develop stereotypies when faced with insoluble problems (Mason, 1991). All these examples appear to be more or less related to (the perception of) uncertainty. While RSBs may initially be driven by such external factors, i.e., over- or underwhelming environments, they may subsequently become independent and self-reinforcing (Mason, 1991). Note that from an evolutionary perspective, both impoverished and overwhelming environments should be surprising to an organism.

In summary, both external environmental as well as internal neurobiological factors can precipitate RSBs, and this general picture fits with the suggestion that basal ganglia feedback relates to RSBs via altered *awareness* of external events (Mason, 1991). Such an awareness should be linked to both, objectively volatile environments as well as alterations of the internal mechanisms for assessing environmental uncertainty. Thus, basal ganglia function appears to take a central role in perceived uncertainty, according to the theory of the brain as a predictive organ.

## Repetitive phenomena in autism spectrum conditions, obsessive-compulsive disorder, and Parkinson’s disease

We next discuss three exemplary conditions, ASC, OCD, and PD, highlighting the expression of RSBs, the role of basal ganglia and dopamine, and predictive processing accounts. We follow our suggestion that neuropsychiatric conditions with basal ganglia involvement may be stratified according to high or low gain of sensory information relative to prior existing knowledge or models (Figure 2). Accordingly, low basal ganglia feedback gain would be characterized by an internal bias, rigid existing models, or strong attractor states and trajectories (Durstewitz et al., 2021), that are excessively hard to break out from (“prior-weighted”). In contrast, high basal ganglia feedback gain would be characterized by relatively unreliable prior models, and/or by placing disproportionate weight on new perceptual evidence (“data-weighted”) (Palmer et al., 2015a). The latter would result in excessive salience, indiscriminate widespread affordances, and high environmental uncertainty. As suggested in the literature for the case of ASC (see below), these individuals might respond to perceived chronic high environmental uncertainty by actively restricting their behavior to detail and repetition, in an attempt to increase the predictability of sensory perceptions. This active focus supports environmental niches within which the agent can operate with relative certainty (Constant et al., 2020). In this way, stereotyped behavior resembles a compensatory form of active inference in pursuit of predictable environments. Conversely, stereotyped behavior in individuals affected by OCD, while sometimes superficially similar, might instead be the result of overly precise prior beliefs (Levy, 2018). In this case, perceptual evidence will be sampled repeatedly in order to best approach excessively precise model expectations. PD occupies a unique position because basal ganglia feedback, and hence the bias across the internal-external spectrum, may fluctuate drastically depending on disease progression, medication, or surgical treatment. Accordingly, ON or OFF states in PD would precipitate systematic changes in the weighting or gain of sensory signals and action opportunities, although this effect may be partly clouded due to chronic long-term treatment and plastic changes.

### Autism spectrum conditions

Autism spectrum conditions (ASC) is an umbrella term for a range of increasingly diagnosed neurodevelopmental conditions (1−2% in high-income countries) of varying expression (Sharma et al., 2018). According to current diagnostic criteria, ASC is characterized by atypical communication, social interaction, and perceptual processing, as well as intense interests and repetitive behaviors. Intellectual and language impairments often co-occur, and ASC individuals may additionally suffer from psychiatric comorbidities such as anxiety, depression, ADHD, or bipolar disorder (Sharma et al., 2018). In a presentation with mild or absent intellectual disabilities (often termed “high functioning” or “low support needs”), autism is increasingly seen as an expression of natural human neurobiological variation that should not be considered as a disorder or disease *per se* (Jaarsma and Welin, 2012; Hens et al., 2019). For this reason, we are using the term ASC here, instead of the official DSM-5 term, autism spectrum disorders (ASD). Individuals with autistic traits can be reliant on routines and rituals and can display a strong focus on highly specific topics and detailed activities, a propensity that can bring advantages in certain professions such as academic work and occupations requiring strong pattern recognition abilities (Wei et al., 2014; Austin and Pisano, 2017). Autistic individuals may find it difficult to generalize from specific knowledge, often have concrete and literal thinking styles, have a limited ability to see the “big picture,” are hyper- or hyposensitive to sensory input, and show enhanced attention to, and discrimination of, simple over complex stimuli. They may also show impaired executive functions (Johnston et al., 2019) such as difficulty planning and organizing activities, or switching their attention between tasks. Especially with strong monotropic interests or phases of hyperfocus, switching from a task or topic of current attention to a different one may be challenging (Murray, 2018). Unpredictable environments, particularly ambiguous signals in social settings and language, can induce unease and anxiety. Consequently, a structured, organized, and organizing environment is generally preferred and actively searched for by the individual. Established schedules allow understanding and anticipating activities and expectations. This preference for predictability and regularity as well as the prevalence of repetitive movements (RSBs for self-stimulation and -regulation, so-called “stims”) make ASC an especially interesting case study on the topic of repeating patterns (Crespi, 2021).

### Obsessive compulsive disorder

Obsessive compulsive disorder (OCD) is characterized by intrusive thoughts, images, ideas or impulses as well as an urge to perform certain, often highly repetitive and stereotypical actions. Affected patients might realize the irrational nature of their symptoms yet report substantial loss of control. Compulsive behaviors may be accompanied by strong urges to act out aberrant impulses, which may consume a substantial amount of time of daily activities. The disease—especially in severe forms—leads to a state of constant suspension, strong feelings of unrest and anxiety as well as high levels of depression. About 30% of all patients with OCD have a concurrent major depressive disorder (McNally et al., 2017). About 15% of all patients suffering from OCD have a lifetime history of suicide attempts. Epidemiological data show a 1-year prevalence of 1 to 2% and a lifetime prevalence of 2 to 3% (Ruscio et al., 2010; Stein et al., 2019). Severe forms of OCD typically manifest during adolescence (Stein et al., 2019). Amongst the most prevalent comorbidities are anxiety disorders (75%) and affective disorders (65%) as well as impulse-control disorder, psychotic disorders, and substance abuse (Stein et al., 2019). Interestingly, compulsions occur within certain behavioral domains such as checking, washing, counting, repeating, and aligning of objects. Certain obsessions co-occur with these domains, for example, fears of contamination or bacteriophobia come with washing compulsions. Harm or harm prevention related obsessions co-occur with checking compulsions. Obsessions with symmetry go along with an urge to put things in order. Patients with OCD exhibit impairments in goal-directed behavior, reduced cognitive flexibility and a reduced capability of inhibiting behaviors (Chamberlain et al., 2008, 2021; Robbins et al., 2012; Martoni et al., 2018). Hence, in several cognitive domains there are impairments in adapting and changing behavior.

### Parkinson’s disease

Compared to ASC and OCD, where RSBs are central diagnostic features, repetitive behavioral patterns receive less attention in Parkinson’s disease (PD). In this section, we want to highlight that RSBs are not only common in PD, but also differ in expression depending on disease stage and chronic dopaminergic treatment. The characteristic motor abnormalities in PD are not themselves considered as stereotyped repetitive behaviors, but the underlying neurobiology might nevertheless be related at the basal ganglia level (Langen et al., 2011). In pre-morbid PD, a typical (albeit controversial) personality type has been described as cautious, inflexible, introverted, harm avoiding and low novelty seeking (Luca et al., 2018). Untreated PD patients frequently display set-switching impairments, behavioral rigidity, perseveration, uniform inflexible motion, and obsessive traits (Alegret et al., 2001). There is also a high prevalence of obsessive-compulsive personality disorder (OCPeD) in *de novo* PD patients, characterized by a pattern of orderliness and perfectionism at the expense of flexibility and openness (Nicoletti et al., 2013, 2015; Luca et al., 2018). Similarly, progressive supranuclear palsy, an atypical Parkinson’s syndrome with poorer response to dopaminergic treatment, shows perseveration and perfectionism (Schrag et al., 2010).

A study investigating repetitive speech phenomena in PD found that 28% of all study participants, and about half of those with advanced PD, showed such symptoms (Benke et al., 2000). These fell mostly into a hyperfluent type resembling palilalia, and a dysfluent, staccato-like type resembling stuttering. Both types appeared to a similar extent in ON and OFF states, reminiscent of analogous motor phenomena in advanced PD, such as freezing, motor blocks and festination (Benke et al., 2000). Repetitive speech phenomena can appear not only in idiopathic PD, but also following basal ganglia lesions and in postencephalitic PD cases (Benke et al., 2000).

Long-term dopaminergic (over-)medication increases the risk for impulsive and compulsive behaviors with repetitive and excessive characteristics, such as compulsive shopping or eating, hypersexuality, gambling, and excessive use of dopaminergic medication (Averbeck et al., 2014; Weintraub and Claassen, 2017). Impulsive-compulsive behaviors in PD might also include enhanced artistic creativity (Joutsa et al., 2012; Inzelberg, 2013; Lhommée et al., 2014; Lauring et al., 2019a,b). Curiously, individuals with gambling addiction prefer games requiring monoform repetitive movements, such as slot machines or scratch cards, and sometimes engage in complex rituals for increasing their luck (Averbeck et al., 2014).

The PD-associated phenomenon of punding is characterized by an intense preoccupation with complex, seemingly purposeless, ritualistic, repetitive occupations, and is described as disruptive and unproductive even when goal-oriented (Evans et al., 2004; O’Sullivan et al., 2007; Fasano et al., 2008; Averbeck et al., 2014; Beaulieu-Boire and Lang, 2015). Punding has first been described in stimulant overuse and is related to amphetamine-induced stereotypies. In PD, it is associated with levodopa replacement therapy, and in particular, dopamine dysregulation syndrome (Evans et al., 2004). In contrast to OCD, punding is not associated with distressing intrusive thoughts or fears. Rather, punding activities are often experienced as soothing and calming, but interruption can cause irritation and anxiety (O’Sullivan et al., 2007; Averbeck et al., 2014). According to one review, the prevalence of punding varied between 0.34 and 14% (Spencer et al., 2011), but is often hidden or unnoticed, and therefore likely underreported (Evans et al., 2004; Spencer et al., 2011). Specific punding activities typically depend on to the individual life-history, personal interests, and existing hobbies, but are exaggerated, disruptive, and consume substantial amounts of time. Punding has also been observed in relation to artistic expression (see e.g., Lhommée et al., 2014). They can manifest, for example, in collecting and ordering small objects, but also in creating artworks such as drawing or sketching, including the production of repeated variations of the same features (O’Sullivan et al., 2007) (Figure 3).

## Basal ganglia involvement in autism spectrum conditions, obsessive-compulsive disorder, and Parkinson’s disease

### Autism spectrum conditions

In ASC, structural magnetic resonance imaging (MRI) findings suggest changes in striatal volume correlating with repetitive behaviors (Hollander et al., 2005). Also, a relationship has been reported between circumscribed interests and the volume of the nucleus accumbens and orbitofrontal cortex (Langen et al., 2014; Yerys, 2015). Evidence from one study suggested a functional underconnectivity of long-distance cortico-cortical connections based on functional MRI measurements, particularly involving regions associated with Theory of Mind (Kana et al., 2009). Another functional MRI study compared the interaction strength between cortical and subcortical resting-state networks in ASC and control subjects. This study found increased functional connectivity between thalamic and basal ganglia networks with cortical primary sensory networks in the ASC group (Cerliani et al., 2015). Resistance to change has been related to atypical sensory processing. A functional MRI study in autistic children found hypersensitivity to novel auditory stimuli seen by an increased activation of motor and sensory cortical regions in an oddball task, but also better performance due to faster reaction times (Gomot et al., 2008). The “Intense World Theory” of autism (Markram and Markram, 2010) proposes hyper-functioning of local neuronal microcircuits leading to excessive perception, attention, memory, and emotionality, which together elicit overly strong responses to experiences. In an attempt to avoid excessive stimuli and surprise, individuals would then restrict their behavior and attention to narrow, detailed aspects of an otherwise painfully intense world.

The molecular mechanisms of ASC are still insufficiently understood, but seem to include alterations in many different neurotransmitter systems (Marotta et al., 2020). The dopamine hypothesis of ASC proposes that autistic behavioral traits may arise from changes in the midbrain dopaminergic system. Especially atypical functioning of the mesocorticolimbic and nigrostriatal pathways are assumed to contribute to social reward alterations and repetitive movements, respectively (Pavăl, 2017; Pavăl and Micluţia, 2021). Pharmacotherapy (when used in addition to behavioral/environmental interventions) may include dopamine antagonists such as atypical (but also typical) antipsychotic agents (Hellings et al., 2017; Eissa et al., 2018). In line with this clinical experience, a mouse study implicated increased striatal dopamine function in autistic-like behaviors (Lee et al., 2018). Also, a *de novo* mutation of the dopamine transporter, favoring dopamine release rather than reuptake, has been identified in whole-exome sequencing of ASC families (Hamilton et al., 2013; DiCarlo et al., 2019). These findings fit well with a central role of the striatum in ASC, and more generally, of the basal ganglia at the interface between organism and environment (Fuccillo, 2016). In contrast, other studies found reduced markers of dopaminergic function in ASC mouse models (Chao et al., 2020), favoring insufficient dopaminergic function in ASC. Additionally, the proposed beneficial role of oxytocin in ASC has been associated with its role in facilitating dopaminergic transmission in the mesocorticolimbic pathway (Pavăl and Micluţia, 2021). The relevance of differences in the dopaminergic system also appears plausible considering the high degree of overlap between ASC and attention deficit hyperactivity disorder (ADHD) traits (Koi, 2021) and the reported importance of interest and motivation for sensory processing and executive function in ASC (Murray, 2018). While an exhaustive review is beyond our scope here, these examples highlight that many findings concerning dopamine function in ASC are still conflicting and requiring further research.

### Obsessive compulsive disorder

Brain regions involved in the neuropathophysiology of OCD are orbitofrontal regions, the ventromedial prefrontal cortex, the basal ganglia and cortico-striato-thalamo-cortical (CSTC) loops (Whiteside et al., 2004; Chamberlain et al., 2008; Menzies et al., 2008). In terms of basal ganglia dysfunction, aberrant activity of CSTC loops is one of the most consistent findings in OCD (Peters et al., 2016). Thereby, elevated activity of the caudate nucleus during habit performance is closely related to compulsivity. Dysfunctions in the subthalamic nucleus (STN) are involved in impairments of inhibitory processes. The right middle frontal gyrus has been connected to stopping behaviors. Involvement of these regions in OCD may explain the inability to withhold compulsive behaviors or repress obsessive thoughts. Similar to PD, deep brain stimulation of the subthalamic nucleus is an effective treatment option, when stimulating fibers analogous to the hyperdirect pathway originating from certain prefrontal cortical areas (Li et al., 2021). This suggests an analogously insufficient basal ganglia feedback in PD and OCD, albeit with a predilection for different functional systems.

### Parkinson’s disease

Parkinson’s disease is most clearly associated with basal ganglia function. PD is a complex disorder of motor and non-motor systems resulting from characteristic degeneration of dopamine-producing neurons particularly in the substantia nigra pars compacta, which innervate medium spiny neurons in the striatum. Furthermore, PD also affects non-dopaminergic neuromodulatory systems (Huynh et al., 2021; Weintraub et al., 2022). In prodromal PD, non-motor symptoms such as autonomic dysfunction, REM-sleep behavioral disorder, depression, or loss of smell, may already be present (Zis et al., 2015; Berg et al., 2021). The slow neurodegenerative process precedes the classical motor symptoms of bradykinesia, rigidity, rest tremor, reduced movement amplitudes, shuffling gait, and postural instability. Dopamine replacement therapy and deep brain stimulation in the subthalamic nucleus or internal globus pallidus both exert their therapeutic effects through enhancing basal ganglia feedback (Elkouzi et al., 2019).

## Predictive processing accounts of autism spectrum conditions, obsessive-compulsive disorder, and Parkinson’s disease

### Autism spectrum conditions

Autism spectrum conditions has been described in terms of active inference (Palmer et al., 2015b,^2017^). Active Inference is a motor-sensory implementation of predictive processing, by which active behavioral selection maximizes observations that align with prior expectations. In the case of ASC, this process might be repurposed for behavioral restriction, where sensory exposure is restricted to manageable and predictable patterns. This behavioral adaptation is interpreted as a response to perceived indiscriminate and overburdening uncertainty, and as such, would favor repetitive activities due to their higher predictability (Lawson et al., 2014, 2017; Palmer et al., 2015b,^2017^). Conversely, situations that are inherently complex, erratic, and uncertain will be avoided, and this particularly includes social situations, where interpreting complex signals about the mental states of other people may be overwhelming. However, atypical sensory weighting and subsequent active environmental sampling are highly context dependent in ASC and may vary depending on type of stimulus, environmental complexity and individual factors (Palmer et al., 2017).

The aberrant precision account of ASC (Pellicano and Burr, 2012; Lawson et al., 2014) proposes an explanatory framework for atypical sensation and perception in ASC informed by Bayesian models. Pellicano and Burr (2012) suggest the formation of so-called hypo-priors that may lead to more accurate perception and reduced reliance on prior experience. This view has been used to explain sensory and other non-social features of ASC, such as repetitive movements and self-regulating behaviors (stimming) as means to reduce environmental uncertainty. Several empirical studies on neurocognitive mechanisms, neuromodulatory hormones and perceptual processing have generated results supporting an imbalance of the precision ascribed to sensory evidence relative to prior beliefs in ASC (Lawson et al., 2014). This overall tendency toward reduced generalization may thus lead to constrained motor plans and perceptual sensitivities but has also been suggested to reduce an individuals’ reliance on heuristics and cognitive biases (Rozenkrantz et al., 2021). However, other recent studies suggest that structural and contextual priors may be intact in ASC (Croydon et al., 2017; Manning et al., 2017; Constant et al., 2020), which calls into question the validity of the proposed hypo-priors.

A comparable predictive processing account has also been hypothesized for the social aspects of ASC (Palmer et al., 2015b). Here, the authors have attributed differences in social cognition between autistic and neurotypical individuals to a diminished set of counterfactual predictions and reduced perceptual presence of others’ mental states in ASC.

More recently, the hypothesis of high, inflexible precision of prediction errors in autism (HIPPEA) has attempted to summarize the social and non-social aspects of ASC within a predictive processing framework (Constant et al., 2020). Rather than identifying specific dysfunctions, HIPPEA aims to trace ASC traits to differently “tuned” general neurocognitive mechanisms. Similar to aberrant precision accounts, HIPPEA proposes that ASC leads to atypically high precision assigned to bottom-up prediction errors. This in turn will lead to overfitted models that will not readily generalize to new inputs. Interestingly, the finding that ASC individuals tend to be less susceptible to perceptual illusions (Palmer et al., 2013; Lawson et al., 2014; Gori et al., 2016) supports the notion of overfitting for incoming sensory stimuli in the moment, rather than relying on existing models based on regularities from experience. From an ecologically informed perspective, the HIPPEA framework describes sensory and social avoidance, reliance on routines and sameness, and reduction of novel stimuli as means to construct a predictable sensory niche for the autistic person in otherwise complex and overwhelming environments (Constant et al., 2020).

### Obsessive compulsive disorder

Predictive processing has been applied to OCD, although less extensively compared with ASC. These accounts generally share a presumed imbalance between top-down predictions and incoming sensory data (Levy, 2018). Moore (2015) proposed that the experience of the world being “not just right” stems from a mismatch between (counterfactual) narratives at global and sublinguistic inference. This causes, among other things, an intolerance for uncertainty, threat beliefs, or fear of causing harm, leading to compulsions as an attempt for correction. The so-called REBUS model (“relaxed beliefs under psychedelics”) suggests that a range of conditions, including OCD, might be characterized by overweighted prior beliefs. These “top-heavy” high-level models could be prioritized over sensory data, such that the rich information contained in lower hierarchical levels is relatively underweighted (Carhart-Harris and Friston, 2019). An example of an overweighted prior could be an anticipated threat, leading to repeated sampling of the world that is nevertheless insufficient to meet excessive prior beliefs. An intriguing observation by individuals receiving deep brain stimulation surgery for OCD is an increase in self-confidence, i.e., the sense of power to act in the world, and that actions lead to expected outcomes (Kiverstein et al., 2019). The authors implicated DBS-induced changes in precision expectations, rendering individuals more receptive to relevant action opportunities (i.e., affordances). Similar to PD, deep brain stimulation enhances the feedback function of the basal ganglia (Li et al., 2021). Interestingly, when stimulation parameters are too high, behavior can become more impulsive, which could be interpreted as excessive self-confidence (Kiverstein et al., 2019). These DBS-related observations also fit well with the notion that a heightened selection and reinforcement mechanism improves the detection of what external events are caused by an individual, and hence, the sense of agency (Redgrave et al., 2011).

Is it possible to find hints for this view in creative expression and aesthetic experience? A higher preference for visual symmetry has been noted in OCD (Summerfeldt et al., 2015). In a detailed, first-person, art-based exploration, a case was made for a relation or even equivalence between artistic expression and the experience of OCD. This account emphasized topics of responsibility, fear of disaster, control and doubt (Baugh, 2015). One can get the impression of how an urge to achieve a “just right” feeling could facilitate artwork that depicts symmetrical, flawless, and even prototypical versions of the world, at the possible expense of variability and detail. This may be interpreted as a top-down perceptual mode that is underappreciative of detail (Figures 2, 4).

### Parkinson’s disease

**FIGURE 7:**
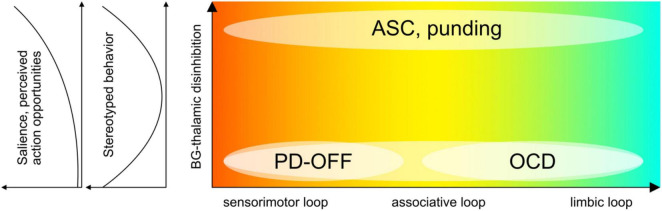
Externally versus internally weighted dynamics shown against the different parallel basal ganglia loops. Stratification of stereotyped behaviors along parallel cortico-basal ganglia loops (*x*-axis) and level of thalamic disinhibition by basal ganglia output (*y*-axis). Lower BG-thalamic disinhibition corresponds to behavior determined more by top-down, prior models. This putatively shapes phenomena in OCD and PD-OFF. Note that beyond motor symptoms, PD-OFF can show severe cognitive and affective alterations as well. Higher BG-thalamic disinhibition corresponds to higher weighting of bottom-up sensory data, by disinhibiting thalamic matrix cells and flattening of the attractor landscape of cortical state space, thereby reducing state change resistance (Shine, 2020). Higher BG-thalamic disinhibition putatively shapes phenomena in ASC and punding.

Due to its neurochemical and neurophysiological alterations, PD offers a unique perspective for linking predictive processing with underlying brain structure. Briefly, one hallmark of PD are abnormally synchronized oscillations in the beta frequency band (around 15−30 Hz) in the basal ganglia system (Silberstein et al., 2003). This synchronized activity is coherent between motor cortical areas and the ipsilateral STN and is suppressed by deep brain stimulation (Oswal et al., 2016). In the canonical cortical microcircuit model of predictive processing, activity in the beta frequency band is associated with deep cortical layers carrying top-down predictions (Bastos et al., 2012; Friston et al., 2015). Because synchronized activity relates to synaptic gain (Friston et al., 2015), this appears to show in a very concrete and measurable way that there is an asymmetry in bottom-up (prediction errors) and top-down (predictions) signaling in PD, such that the PD-OFF state falls on the side of relatively overweighted top-down priors propagated through the cortico-basal-ganglia system (see Figures 5, 7). On the phenomenological level, in contrast to the rigid and harm-avoiding pre-morbid PD personality type, long-term treated PD patients with impulsive-compulsive behaviors generally show increased novelty seeking, information sampling, and temporal discounting in neuropsychological tests. Intriguingly, these differences could be accounted for by an increased uncertainty about future rewards and information (Averbeck et al., 2014). Another empirical study also found a connection between impulsivity in PD patients and increased subjective environmental volatility, with a possible role for the subthalamic nucleus in the modulation of outcome certainty (Paliwal et al., 2019). Following the addiction hypothesis that dopamine increases the salience of reward-related stimuli, this fits well with the close connection in predictive processing between increased salience (prediction errors) and higher perceived uncertainty.

In summary, behaviors with a compulsive and stereotypical component can be seen in different stages of PD, including untreated and long-term (over-)medicated individuals. What distinguishes these phenotypically partially overlapping behaviors? We suggest that in PD, chronic alterations in dopaminergic tone, and subsequently basal ganglia feedback gain, precipitate the appearance of a more obsessive-compulsive-like set of stereotypies in pre-morbid and untreated PD patients, whereas an autism-like set of RSBs is associated with long-term dopaminergic medication. This could mean that the aberrant precision account of ASC, informed by predictive processing, would similarly apply to punding in PD patients.

### Extending the tripartite model

Based on these differing predictive processing accounts, we suggest an extension of the classical tripartite model of basal ganglia associated RSBs (Figure 7). In addition to a stratification according to the separate basal ganglia loops, the respective generic selection and reinforcement function may be over- or underactive. Similar to the well-known motor and motivational reinforcement function of basal ganglia feedback, this brain structure might also influence the weighting of affective, cognitive, and sensory signals, thus affecting their salience, which correlates with (perceived) uncertainty. The overall consequence of either too much or too little basal ganglia reinforcement may be an increased likelihood of RSBs. If it holds true, this distinction is highly relevant because it would imply contrasting mechanisms that require adjusted management approaches, even in cases of highly similar RSB phenomenology.

## Aesthetic learning and basal ganglia function

Aesthetic learning is the acquisition of perceptual, affective, and cognitive knowledge through interaction with cultural artifacts and art—a branch of research that has lately received more and more interest (Pelowski et al., 2016). Via aesthetic learning we gather information about ways of engagement that can be described as artifactual habits, that is: we have become attuned to images, artworks, and other artifacts, and the action-perception loops they afford (they can be described as “designer environments” in Clark’s understanding, see Fingerhut, 2021). Aesthetic objects have their own sets of affordances, and humans learn the specific ways for gathering epistemically relevant sensory information, i.e., information that helps to optimize behavior by balancing internal expectations and external signals (Friston, 2009; Kesner, 2014; Friston K. J. et al., 2017; Van de Cruys, 2017).

This kind of attunement might be altered in people with basal ganglia conditions (Figures 2, 7). In order to understand the potential of aesthetic learning as a tool for therapeutic interventions (especially for the phenomenon of RSBs within different kinds of neuropsychiatric disorders), we need a framework allowing the concept of active inference that addresses the role of sensorimotor integration and its influence on sampling sensory information. This is achieved by respecting not only internal effects but action-perception loops allowing the amplification of either prediction errors (increase of external cues due to high salience) or gaining insight (focusing on internal update of—potentially rigid repeating—generative models).

Interestingly, repetitive behavior described in the neuropsychiatric conditions above might also manifest in artifactual habits. However, they appear more rigid, lacking some of the characteristic flexibility and adaptiveness of the “expansive habits” of neurotypical individuals (i.e., their expansiveness with respect to time, space, and the sphere of activity they afford; Fingerhut, 2020, 2021). While purportedly increasing predictability, repetitive behaviors are insufficient for knowledge gain: it appears that habitualized behavioral routines miss the central point of learning or enrichment. In order to “break” such aberrant and rigid behavior, aesthetic learning may facilitate experiences that are intense enough (experiences of high affective value) to help elicit novel behavior conceptually and spatiotemporally adapted to the environment, that is, usable for future predictability.

The reward and appreciation of aesthetic experiences have especially been associated with dopamine activity and novelty-seeking in both, research in predictive processing (Kesner, 2014; Friston K. J. et al., 2017; Van de Cruys, 2017) and in art and aesthetics research (Spee et al., 2018). Here, the aesthetic triad of semantic, evaluative and affective dimensions of aesthetic experience might roughly relate to the tripartite model of associative, sensorimotor, and limbic basal ganglia circuits (Langen et al., 2011; Yerys, 2015; Spee et al., 2018). Dopamine also plays a role within the predictive processing framework by enhancing action-relevant stimuli (Friston K. J. et al., 2012). Although dopamine modulates cortical networks as well, it is a particularly important driver of basal ganglia feedback loops by selectively disinhibiting thalamocortical neural projections. In this way, dopamine-dependent basal ganglia output to the thalamus appears ideally suited to modulate sensory perception. Referring back to Section 2, basal ganglia appear to be at the interface of an organism and the environment, expanding a landscape of affordances. Stimuli such as artwork seem to amplify this, but with the prospect of a directed learning component regarding personal and contextual values. Furthermore, the basal ganglia control the coordination of ongoing activities and behaviors, but also the switch between them. Again, aesthetic objects seem to facilitate this due to salient features, also considering that the space of engagement is often perceived as safe (increased predictability at a contextual level). An artistic space, thus, allows developing new perspectives, and modulation or creative expression of repetitive behaviors, depending on how they are perceived by the individual.

## Aesthetic learning in repetitive stereotyped behaviors

What role can aesthetic learning have in RSBs? RSBs are strong habits that enhance predictability and thereby safety. However, engaging with aesthetic objects under the umbrella of aesthetic learning appears to be connected to expansive habit formation with epistemic value (Fingerhut, 2020, 2021). This value might enable initiating action-policies in favor of optimizing environmental adaptation. Depending on individuals’ necessities and needs, together with the appropriate selection of personally relevant artifacts, high salience signals can be fostered and/or new information gathering spaces can be created. This kind of engagement thereby allows the exploration of the whole spectrum of external and internal signals (see Figure 2), enabling normalization of either data-weighted or prior-weighted behavior. Aesthetic learning might provide such amplification reaching to the endpoints and pulling behavioral patterns into accurate behavior (Sarasso et al., 2021), by creating conducive environments and learning contexts for action and experience (Wickman, 2012). It uniquely bridges external events and artifacts with inner feelings and therefore may help to align external realities with internal generative models. This process is not restricted to art but is continuous with all life experiences (Wickman, 2012). As such, aesthetic learning may help to form experiential habits, rules and norms suited to make use of a larger stretch of this salience/affordance spectrum. While certain external realities with strong valence may not be changeable, one may at least learn to alter one’s sensory and bodily involvement in a given context. This means that aesthetic learning can either provide structured learning environments or allows getting used to objects or situations by learning how to adjust one’s experience.

Abstract art can also support the updating of internal beliefs about figurative pattern assemblies (Gestalt perception) (Van de Cruys and Wagemans, 2011a). Going back to the visual arts, both impressionism and expressionism can also address rather content-specific aspects and their affective value, and update the emotional meaning encoded in generative models. Aesthetic learning (as an activity in a safe space) enhances cognitive as well as affective updating of both, sensorimotor patterns and generative models. Generally speaking, by actively seeking out and resolving uncertainty, a person thus feels more confident in his/her behavior (Friston K. J. et al., 2017). This falls into the already well-established discussion of the predictive processing framework in learning (Friston, 2009; Schwartenbeck et al., 2013; Tik et al., 2018). Most importantly, aesthetic learning might balance new sensory information with a multiplicity of prior (veridical and counterfactual) models. Consequently, firmly anchored prior beliefs may be opened to new perspectives, or conversely, input of too many different stimuli with excessive salience may be focused more toward those with the highest epistemic value. Hence, aesthetic learning and using engagements with aesthetic objects might help to balance internal (agentic) and external (environmental) signals.

## A role for aesthetic learning in autism spectrum conditions, obsessive-compulsive disorder, and Parkinson’s disease: heightened prediction error acceptance and insight

So far, we have shown examples of how aesthetic experience and creative expression might be shaped by characteristic perceptual modes spanning between novelty and familiarity in ASC, OCD, and PD (Figures 3, 4). But how could these regularities apply to creative art and occupational therapy? Also, are there empirical therapeutic practices that already fit into this framework? At this point it is important to note that the categorical perceptual modes depicted here−proneness to novelty, detail, and surprise versus an expectation-driven tendency for generalization−are unlikely to be mutually exclusive and should not be seen as an overly unified picture. Instead, they probably exist in parallel across sensory modalities and levels of abstraction, with considerable variability due to psychosomatic state, personality type, personal and socio-cultural background, experience, preferences, motivation, affective style, contextual priming and social situation. All of these factors together tune the predictive apparatus toward salient objects and experiences (Kesner, 2014). For example, the tolerance for surprising experiences might be increased in predictable settings (i.e., settings that are experienced as safe of known), whereas novel sensations in one sensory modality might be less tolerable in the presence of concurrent unexpected sensations in other modalities. Although we argue for characteristic cognitive styles in tolerating and resolving ambiguity, as well as in recognizing and categorizing perceptions, such cognitive styles are not necessarily an expression of pathology. Indeed, they could become advantageous and a source of inspiration in permissive contexts. This might be particularly true when engaging in artistic and creative expression (see e.g., the work of Yayoi Kusama characterized by obsessions and repetitions, Ferrell, 2015). Here, we see the value of aesthetic learning within a safe art therapy space in the dynamic expansion of the tolerable surprise-predictability spectrum, and in how well this active-perceptual skill can be transferred to everyday life circumstances. Within the constraints of neuropathological processes, an individual could learn to adjust own expectations and also to tolerate or even modify those exposures and situations that resist immediate understanding (Kesner, 2014). This account links well to agency and self-actualization, increasing confidence and coping mechanisms as well as cognitive and social functioning (Waller, 2006; Schweizer et al., 2014; Chiang et al., 2019). Through aesthetic learning, artifacts that are socially and culturally classified as aesthetically valuable can be used as an attention magnet, allowing heightened and prolonged prediction errors (Van de Cruys and Wagemans, 2011b; Kesner, 2014; Van de Cruys, 2017). Conversely, “outsider art,” beyond the boundaries of official culture (or deliberately violating it), may sometimes be more suitable to accommodate characteristics attributable to particular conditions (Roth, 2020).

Objects or sensations that are personally preferred, beautiful or aesthetically appealing, or even thought-provoking, irritating, or shocking, are thus more salient than other sensory inputs. This attention amplification could serve to regulate distressing repetitive behavior and transition into new action-perception cycles. Some art examples, which probably lead to good responsiveness here, were also presented in a recent review (Seth, 2019). One example is the enhancement of focal vision and peripheral blurriness by rendering the periphery ambiguous and the central vision unambiguous. Other anchor points would be especially salient coloring, objectification, or artistic ways of reduction. This is reminiscent of attention-focusing clinical applications such as cueing devices for individuals with PD. Such applications, focusing attention through saliency, can help to overcome motor symptoms such as gait freezing. It could be speculated that aesthetic appeal (respecting the social-cultural space and personal preferences) might amplify the effect of such measures beyond the immediate cue/stimulus. Relatedly, case evidence suggests that individually selected music can support treatment of gait impairments (Holter et al., 2022), and music may provide better affordance than simple metronomes for gait cueing (Rodger and Craig, 2016).

We hint here at just a few concrete examples of how the present framework may integrate with creative art therapy, offering inroads for future work. Visual art in particular has inherent representational, symbolic, and abstractive qualities. Thus, in autistic individuals, producing artistic expressions may improve abstract thinking and the ability to generalize (Alter-Muri, 2017), while at the same time allowing to employ the aesthetic qualities of likeness and repetition (Roth, 2020) for mentally ordering the multifaceted complexity and intricacy of worldly experiences by bringing them on paper. Both of these aspects should help to develop skills for regulating sensory over-/understimulation (Alter-Muri, 2017). Since sensory withdrawal in response to overwhelming sensitivity may impair social attachments in ASC, such skills may secondarily improve the capacity to communicate and attach to others (De Jaegher, 2013). Moreover, art making becomes an additional non-verbal communication channel: the art object exists in the space between creator and viewer/therapist, providing a learning experience for both (Waller, 2006). Similar to stimming in ASC, which increases the salience of one stimulus over many competing ones, making visual art may help to focus attention and regulate hypersensitivity to subtle environmental changes−thus using artistic elements to guide perceptual experience (Seth, 2019). Both motor mannerisms and focusing on a created object may similarly help to modify stimulation overload (Alter-Muri, 2017).

As a concrete example from OCD, we point to the artist Yayoi Kusama, who, by “repeatedly incorporating her fears into her works,” recreates a form of exposure therapy for the fears underlying her obsessions and compulsions. In contrast, another side of Kusama’s work rather represents purity and control of her *surroundings* (Ferrell, 2015). Thus, her lifelong experience with OCD and creative expression apparently helps to control her obsessions over her fear (internal expectations), but also her perceptual experiences (external environment). For the purpose of providing therapeutic space for creative expression, our theoretical considerations further suggest that both an orderly, minimal set of materials but also an area for messy experimentation should exist. In both, ASC and OCD, this should allow a person to “get dirty” but also get “every detail accurate,” according to current needs (Waller, 2006).

In PD, it seems relevant that salient signaling through art is capable of both enhancing (being aroused and moved in an affective sense; Pelowski et al., 2017) and inhibiting movement (“stopping for knowledge;” Sarasso et al., 2020a). On the one hand, the absorption in a creative process may distract from motor symptoms and ruminations (Strand and Waller, 2010). On the other hand, the art therapeutic setting may make specific (motor) impairments matter less, fostering instead a sense of ownership and pride (Alter-Muri, 2017). For example, repetitive behaviors and movements “can be channeled through art in socially accepted activities” (Alter-Muri, 2017). While intended at ASC, these principles similarly apply to PD. Additionally, certain activities such as printmaking could accommodate a need for sameness while also circumventing motor symptoms. In PD in particular, art making can gain profound personal meaning, can provide motivation despite physical limitations, and may help to discover new ways of doing things (Wadeson, 2003). This empowering case description concerns an individual who found a new meaningful occupation, allowing him to focus on intricate details, using a sponge if a brush cannot be used, and expressing himself even if he is no longer able to write legibly. Curiously, he notes that when he cannot move in an OFF state, he observes things around him the most (Wadeson, 2003). This sentiment has also been expressed by the artist whose work is shown on the right in Figure 4 (personal communication). Lastly, particularly in those individuals with an enhanced artistic drive (Inzelberg, 2013), art therapy could take some cues from exercise-based behavioral treatments focused on speech and limb motor systems with the goal of increasing voice and motor amplitudes (Fox et al., 2012). In addition to often detailed and miniscule forms of creative expression in PD, large canvases and broad brushes could be useful to calibrate the senses and range of motion, enhancing agency and the ability for self-cueing.

In summary, aesthetic learning can address the dynamic spectrum of agent-environment dynamics. Aesthetic tools and practices can be put to art therapeutic use in support of sensing and sense-making, creative engagement, and cognitive-emotional value. Such interventions can either focus on changing the *perception* of affordances, or they can focus on strategically altering the *environment*. Although we have mostly focused on visual arts, it should be noted that creative art therapies also include, e.g., music, dance, drama, theater, or creative writing.

## Discussion

Repetitive stereotyped behaviors are associated with environmental and neurobiological factors, and may result from idiosyncratic, atypical, or disrupted predictive brain processes within more or less volatile environments. This follows quite simply because an effective behavioral strategy to improve predictability is to engage in repetition (Keller and Mrsic-Flogel, 2018). Given this relationship between prediction and repetition, and the extensive evidence for basal ganglia involvement in RSBs, we hypothesized a role for the gain computation provided by the basal ganglia (akin to a “generic selector;” Redgrave et al., 2011) that is compatible with the predictive processing framework and has implications for perception and aesthetic learning, beyond motor control. An altered predictive capacity might be caused by internal or external factors, i.e., changes in the brain’s predictive machinery or changes in environmental complexity, that together calibrate brain representations (generative models) of self, body, and environmental regularities. The predictive processing literature has produced accounts of several neuropsychiatric disorders. By focusing on RSBs in ASC, OCD, and PD as exemplary conditions, and combining clinical observations, structural and functional subcortical alterations, existing predictive processing accounts of these conditions, and differences in aesthetic experience, we suggest the following key findings that may improve their understanding and management, and lead to testable hypotheses: (i) The basal ganglia are plausibly positioned to modulate the dynamic balance between prior learned regularities and automated behavioral patterns, versus flexible adaptation to new environmental information (Figure 5; Shine, 2020). Thus, we hypothesized that the feedback gain provided by the basal ganglia enhances the saliency of sensory perceptions and action opportunities (affordances), and reduces the threshold for switching behavioral trajectories. This view supports a role for the basal ganglia in perception, in parallel to the reinforcement of movements (Yttri and Dudman, 2018), and is compatible with the close overlap between motor and sensory predictions (Colder, 2015; Parr et al., 2022). (ii) In our model, both overweighted internal prior models as well as abnormally increased sensory precision expectations may lead to RSBs. While the former process compels an individual to repeating model-confirmatory action, the latter causes adaptive strategies to increase perceptual predictability, such as narrow interests and insistence on sameness. This model might better contextualize clinical phenomena such as differing treatment strategies (exposure therapy versus sensory shielding in OCD and ASC), and neurophysiological findings such as different proneness to perceptual illusions or electrophysiological markers of surprise (Pekkonen, 2000; Dunn et al., 2008; Palmer et al., 2013; Ding et al., 2017; Sarasso et al., 2021). (iii) If it holds true, the basal ganglia output should be regarded as a core parameter in all neuroanatomically informed predictive processing models, in line with recent accounts (Shine, 2020), and in contrast to more cortex-centric views (Bastos et al., 2012). (iv) The differentiation between high and low basal ganglia function has implications for creative art therapy in conditions characterized by RSBs and basal ganglia involvement. Behavioral phenomena that appear highly similar, might nevertheless benefit from differing approaches that provide stability or encourage novelty and exploration. (v) Altered predictability may result from environmental changes, i.e., overwhelmingly complex, but also impoverished (particularly social) contexts and stimuli, in addition to how they are perceived. This is underscored by the developmental impact of the social environment on RSBs (Lutz, 2014). Additionally, RSBs can also be linked with altered *awareness* of external events (Mason, 1991). If basal ganglia output influences perceived uncertainty of environmental stimuli through altered salience of action-relevant sensory stimuli, this should particularly impact social signals, as is the case in ASC. Accordingly, basal ganglia disorders cannot be managed in isolation from the environmental and social context of the individual, due to bidirectional causalities between brain disorders and social embeddedness (Dhand et al., 2016). The social context represents a potentially underappreciated determinant in basal ganglia disorders. This is further supported by the evolutionary argument that the dopamine-dominated striatum in humans has favored an outward-oriented, externally driven, cooperative personality type capable of extended social-cultural group formation and shared intentionality (Raghanti et al., 2018). (vi) An interesting neuroanatomical detail is the fact that at the level of the thalamus, input from deep cerebellar nuclei appears to be parsed in a similar fashion as external sensory signals, namely through the core thalamic cells that project to deep cortical layers (Shine, 2020). These specific signals are then modulated by the more diffuse matrix-type basal ganglia feedback. It is plausible to consider excessive amplification of widespread cerebellar signals through overactive basal ganglia feedback in the pathogenesis of dyskinesia, a motor complication in PD treatment characterized by chorea-like uncontrolled movements (Espay et al., 2018). A similar amplification of indiscriminate sensory signals might well resemble a “perceptual vigor” up to “dyskinesia of the senses,” but would be much less obvious to the observer, compared to visible motor expressions. (vii) If two forms of RSBs exist depending on high/low basal ganglia gain, they might differ in their association with anxiety and stress. The feeling of looming bad outcomes based on fixed prior beliefs in OCD has a different quality than focusing on a preferred narrow repetitive activity in ASC or punding, where behavioral restriction reduces exposure to indiscriminately salient environmental features. (viii) This also questions the face validity of animal models for stereotyped behaviors, and suggests prioritizing construct and predictive validity (Lutz, 2014).

Overall, we suggest that repetitive behavioral and cognitive phenomena could result from overly skewed agent-environment dynamics on an external-internal axis of signal weighting by the basal ganglia. This might resolve some of the conflicting evidence for the tripartite model of RSBs (Yerys, 2015). On top of the stratification of repetitive phenomena according to affected limbic, associative, and sensorimotor loops (Langen et al., 2011), we propose that a second axis should depict the level of thalamic disinhibition by basal ganglia feedback (Figure 7). This might offer an additional explanation for the phenomenological variability within and between neuropsychiatric conditions.

One of the fundamental insights of predictive processing and active inference is to recognize the role of prior expectations in perception and action. Likelihood estimations and preferences of encountering particular sensory information influence action selection. The weighting of error signals resulting from the comparison between actual sensory information and prior predictions influences adaptation to changing environments versus adhering to previously learned sequences, likely through the cooperation of basal ganglia and cerebellar feedback circuits (Shine, 2020). Such a process must provide space for wide variability (biological, contextual, functional, spatial, temporal) in the extent to which new sensory information is weighted relative to prior expectations. If this weighting is skewed strongly to one or the other end of the distribution, adaptive cognitive and behavioral flexibility may be reduced, and RSBs may occur.

### Nosological implications

The axis of high/low basal ganglia feedback gain might help to group basal ganglia disorders in a biologically meaningful way. Hints in this direction come from clinical experience. In terms of management, while classical anti-dopaminergic neuroleptic drugs are the only somewhat efficacious substance class in severe autism (Sharma et al., 2018), anti-dopaminergic drugs are of uncertain value in OCD treatment (Koo et al., 2010; Pittenger, 2021). Conversely, serotonin reuptake inhibitors (SSRIs) are used in the treatment of OCD (Del Casale et al., 2019), whereas the serotonin hypothesis in ASC would caution against this medication in autism (Harrington et al., 2013), and the evidence for effectiveness of SSRIs in ASCs is doubtful (Williams et al., 2013). Deep brain stimulation is efficacious in reducing OCD symptoms (Rappel et al., 2018; Li et al., 2021), but plays no role in ASC management (although see Sinha et al., 2015). Moreover, deep brain stimulation in OCD and PD appears to work in a similar fashion by reducing excessively synchronized activity of the limbic and somatosensory parts of the hyperdirect pathway to the subthalamic nucleus, respectively (Li et al., 2020; Treu et al., 2020). This suggests similar underlying alterations in the respective basal ganglia networks. In fact, the subthalamic nucleus is itself a target in PD, OCD, and also Tourette’s syndrome (Vissani et al., 2019). Most revealingly, despite some overlap in phenomenology, individuals on the autism spectrum benefit from sensory regulation strategies and predictable environments (Hyman et al., 2020), while psychotherapy for OCD rather includes exposure therapy, confrontation, and response prevention (Stein et al., 2019; Pittenger, 2021). PD seems to take an intermediate position: while certain phenomena in PD-OFF resemble OCD symptoms (Alegret et al., 2001; Maia et al., 2003; Harbishettar et al., 2005), punding, which is particularly seen in long-term (over-) treated PD, has similarities with ASC features. In this view, the PD-OFF state is more OCD-like and the PD-ON state is more ASC-like. Additionally, PD has a particular role here because of the strong and highly visible involvement of the motor system in the OFF and ON states, and the drastic fluctuations that can occur due to pharmacological and surgical interventions. This opens up the possibility to extrapolate from sensorimotor afflictions to limbic and associative symptoms that might be functionally comparable but less obvious than overt motor symptoms.

### Implications for aesthetic learning in creative art therapies

For our purposes here, it is worth emphasizing that a core property of aesthetic learning is a capability to playfully set and violate expectations and thus to challenge the neurophysiological model generation and updating process. It is therefore not unexpected that art experience, as well as creative expression, should vary in systematic ways according to more internally versus externally oriented dynamics, i.e., relying more on previously learned routines versus openness to new experience. Using aesthetic learning has been discussed in the light of predictive processing as providing a secure space for therapy. Within such a safe context, exploration and new experiences, guided by interpersonal coupling through a therapeutic relationship, could catalyze the creative destruction of overweighted priors, and the creative construction of new ones (Vaisvaser, 2021). Building on such proposals, we suggest that creative art therapies may serve targeted goals in conditions characterized by more internally or externally biased agent-environment dynamics. This is a possibly more nuanced approach compared to classical cognitive behavioral therapy or habit reversal, and appreciates the adaptive function of repetitive behaviors in response to perceived over- or understimulation in a given context. Accordingly, neither exposure therapy nor sensory shielding will always be adequate, and a safe context should be expected to increase the tolerance for high salience and perceived uncertainty.

### Neuromodulation of top-down and bottom-up signals

Other neuromodulatory systems likely influence the balance between existing priors and new sensory information. For example, the REBUS model (“relaxed beliefs under psychedelics”) suggests that serotonergic signals influence the precision of existing priors, and overweighted prior beliefs were implicated in several psychiatric conditions (Carhart-Harris and Friston, 2019). From this perspective, the serotonin hypothesis of ASC would imply a reduced precision of prior models, and consequently a relatively increased gain of sensory signals. Complementary to this view of altered precision of prior beliefs, we discuss here that the precision weighting of new sensory data is equally important for dynamic and flexible calibration of generative models and resulting behavior. It is tempting to contrast serotonergic weighting of prior beliefs with dopaminergic weighting of new sensory data, with the former possibly modulating cortical representations, and the latter possibly involving basal ganglia feedback modulation of proprioceptive and external sensory signals. Together, the fluctuations and playful action-dependent alterations between models and data, and their relative precisions, would shape the creative sense-making of the world, in different functional domains and levels of abstraction.

### Limitations

The picture we have painted here is an attempt at a high-level explanation of RSBs and their manifestation in aesthetic experience. As such, it brushes over many heterogeneous and idiosyncratic details in behavioral expression, neurophysiology, and individual constellations of experience and brain changes. Our depiction of predictive processing also leaves out the relative precision of different competing models, or the distinction between discrete and continuous predictions. In terms of the neuropsychiatric conditions discussed here, substantial variability and also comorbidity may exist within and between them. This might partly have to do with the way disease categories are influenced by phenomenology, but also with affected functional domains, context, and many other personal and environmental factors.

## Conclusion and perspectives

In this hypothesis paper, we have focused on recurrent patterns of action and perception. Although these are part of normal development, they may also appear as RSBs in various neuropsychiatric conditions. Based on the tripartite model, these often correlate with changes in sensorimotor, limbic, and associative circuits of the basal ganglia. Using the predictive processing framework, active inference, and the concept of affordances, our goal was to relate altered basal ganglia feedback to the phenomenological variability of RSBs. We have discussed how repetition and prediction appear tightly linked to basal ganglia function, and manifest in patterns of creative expression and aesthetic experience. The impact of basal ganglia feedback on, e.g., openness to experience, sensitivity to (social) salient signals, or creativity, is empirically testable between conditions of high/low basal ganglia function, for example in individuals who receive dopamine replacement therapy or deep brain stimulation. A related suggestion has been to employ behavioral tasks in order to test the effect of deep brain stimulation on precision expectations (Kiverstein et al., 2019). The view presented here might also better contextualize compulsive creativity, as well as opening up the possibility to apply predictive processing to other basal ganglia conditions in a similar fashion.

We have attempted to build a bridge between basal ganglia, dopamine, and aesthetic experiences. By combining a neurobiological and predictive processing perspective of aesthetic learning, we have pointed at implications for creative art therapy, although specific recommendations remain to be elaborated and empirically tested.

## Data availability statement

The original contributions presented in the study are included in the article/supplementary material, further inquiries can be directed to the corresponding author/s.

## Author contributions

BS and MT developed the concept and wrote the first draft. All authors contributed to the writing and editing of the manuscript.
